# Hybrid Semantic–Lexical Retrieval for Source-to-CDE Mapping in the NCI caDSR

**DOI:** 10.64898/2026.10.14.758967

**Published:** 2026-10-20

**Authors:** James H. Tanis, Denise B. Warzel, Rakesh P. Khanna, Qingrong Chen, Chunhua Yan, Ravi Velleleth, Matthew T. Nash, Deepali Manathara, Brad B. Biggers, Joyce B. Soares, Daoud Meerzaman

**Affiliations:** 1 National Cancer Institute, NCI Shady Grove, 9609 Medical Center Drive, Rockville, MD 20850, USA

## Abstract

Accurate mapping of local source data elements to standardized common data elements (CDEs) is critical for biomedical data integration but is challenged by short, abbreviated, and heterogeneous source descriptions. We constructed a benchmark of approximately 69,000 expert-linked source-to-CDE mappings from the National Cancer Institute’s caDSR and evaluated multiple bi-encoder models with two-stage fine-tuning. Here we developed a hybrid retrieval system combining semantic bi-encoder retrieval, CDE Match-inspired keyword retrieval, cross-encoder scoring, and supervised reranking. This system achieved Recall@5 ranging from 0.802 to 0.972 across internal and five distribution-shifted holdouts, outperforming the other evaluated methods on five of six datasets and approaching ceiling performance on the sixth dataset (GDC). These results demonstrate the effectiveness and robustness of integrating semantic and lexical evidence for source-to-CDE mapping, supporting improved interoperability and reuse of biomedical metadata across diverse clinical and research contexts.

## 1 Introduction

Organizations such as hospitals, research networks, and clinical studies often collect data using local naming conventions rather than a shared metadata standard. As a result, the same concept may appear under different variable names, labels, or question text across datasets. Mapping these local source data elements (SDEs) to standardized common data elements (CDEs) is therefore an important step for data harmonization, interoperability, and cross-study reuse [1, 2]. SDEs are local metadata fields—variable names, form labels and question text—used in clinical studies, registries and electronic data-collection systems, including EHR-derived workflows; the inputs to this task are therefore metadata labels and question text, not patient-level records. NCI CDEs are the standardized definitions used to keep those fields consistent across trials and studies [2, 3].

In practice, this mapping task is difficult. Human experts ultimately determine the correct match, but manual review is time-consuming and relies heavily on a priori knowledge of NCI CDEs, particularly given the large candidate search space. The production candidate catalog against which queries are ranked contains roughly 63,000 non-retired CDE records. Benchmark construction and production retrieval use different caDSR snapshots, which we describe in Section 2.1. Curators therefore depend on ranking systems to surface likely matches from source-query text derived from SDE metadata. However, source-side queries are often short, abbreviated, heterogeneous in style, and semantically underspecified. As a result, methods based primarily on lexical overlap may miss correct mappings when wording differs between the source element and the target CDE [4, 5].

We constructed a benchmark of approximately 69,000 expert-linked source-to-CDE mappings from the National Cancer Institute’s Cancer Data Standards Repository and Registry (caDSR) CDEs and used it to evaluate bi-encoders, text representations, and two-stage fine-tuning against a production catalog of approximately 63,000 CDE records. For controlled experiments, we used a local Python implementation developed from the service’s supplied source code that reproduces its core rule cascade and scoring logic against a frozen catalog, although minor implementation and data differences can affect individual rankings.

Earlier metadata-mapping systems relied mainly on lexical and terminology-based methods. Mougin et al. mapped source metadata to reference resources using lexical matching and field values [4], while Pathak et al. combined lexical and ontology-based methods across eMERGE institutions [5]. More recently, Fan et al. combined exact, lexical, and embedding-based semantic matching in a semi-automated pipeline for multi-site EHR flowsheet alignment and concept mapping [6], while CDEMapper combined Elasticsearch, GPT-based embeddings, and reranking for NIH CDE recommendation [7]. In parallel, bi-encoders have enabled semantic retrieval when relevant items share few exact terms with the query [8, 9, 10], including biomedical models such as PubMedBERT, SapBERT, and BioSimCSE [11, 12, 13]. Hybrid sparse–dense retrieval can improve over either representation alone [14], and multi-stage systems can apply cross-encoders to rerank merged candidate sets [15, 16].

These developments make bi-encoders a natural candidate for source-to-CDE retrieval, where the correct CDE may express the same concept as the query using substantially different wording. Task-specific and domain-adaptive training have improved bi-encoder representations in other retrieval settings [10, 17, 18, 19], but, to our knowledge, have not been evaluated for source-to-caDSR CDE retrieval. We therefore test whether fine-tuning on expert-linked source-to-CDE pairs improves retrieval.

Dense semantic retrieval alone, however, captures only part of the evidence useful for this task. Bi-encoders capture overall semantic similarity, whereas the correct mapping can depend on fine-grained lexical evidence. An abbreviation, qualifier, identifier fragment, or rare technical term may distinguish otherwise similar CDEs. Lexical methods preserve these cues [14, 20] but can miss correct mappings when the query and target express the same concept using different words [4, 5].

We therefore combine semantic and keyword retrieval, jointly score each pooled query–candidate pair with a cross-encoder that evaluates the query and candidate together to estimate their relevance, and use a supervised reranker to integrate the resulting evidence. We report Recall@5 and related top-K retrieval measures; we do not evaluate precision.

In this study we evaluate bi-encoders for source-to-CDE retrieval in caDSR, compare them with strong lexical baselines, and integrate them into a hybrid ranking system. We construct a benchmark of 69,102 expert-linked source-query/CDE pairs derived from caDSR metadata (68,142 after canonical deduplication and reachable-gold filtering). Retrieval is performed against a separate production catalog of 62,976 non-retired CDE records. We evaluate on an internal test set together with five distribution-shifted holdout sets designed to assess organizational and style-based shift. We study how query and CDE text representation, model choice, and two-stage fine-tuning with hard negatives affect retrieval quality.

Figure 1 provides an overview of the workflow, including benchmark construction, bi-encoder screening and fine-tuning, hybrid candidate generation, cross-encoder scoring, and supervised reranking. In the final system, FT-MPNet and CDE Match-Fuzzy contribute candidate CDEs to a shared candidate pool. For each query–candidate pair in this pool, a supervised histogram-based gradient-boosting classifier (HGBC) integrates features from the bi-encoder, keyword retriever, and FT-MedCPT cross-encoder to produce the final ranking.

The main contributions of this work are as follows:

1. A large benchmark dataset of expert-linked source-to-CDE mappings derived from caDSR metadata.
2. A systematic evaluation of bi-encoders, text representations, and fine-tuning strategies for source-to-caDSR retrieval.
3. A reproducible CDE Match-inspired keyword system, CDE Match-Fuzzy, that improves on the Python approximation to NCI CDE Match used for comparison.
4. A hybrid keyword and semantic retrieval system that outperforms the Python approximation to NCI CDE Match and all other evaluated methods on five of six datasets, while achieving near-ceiling performance on Genomic Data Commons (GDC) within one query of both the official service and its Python approximation.

## 2 Methods

### 2.1 Dataset, task, and evaluation sets

This study combines a caDSR-derived benchmark, three caDSR-derived distribution-shift holdouts, and two separately assembled external evaluation sets. The benchmark, the in-distribution train, validation and test splits, and the Canada Cancer Trials Group (CCTG), Object Identifier (OID) Alternate Name (ALT), and Clinical Data Acquisition Standards Harmonization (CDASH) holdouts were all constructed from the nightly caDSR XML export downloaded on January 12, 2026. The GDC and Cancer Immune Monitoring and Analysis Center (CIMAC) sets were assembled independently of that export and are used only as external evaluations. The retrieval task is to map a source-like query string to its correct CDE in caDSR. A bi-encoder independently encodes the source query and each candidate CDE description as vector embeddings. Candidates are ranked by cosine similarity, which measures the alignment between the query and CDE embedding vectors. Within the caDSR-derived benchmark and its holdouts, query strings were harvested from two metadata sources: alternate names (ALT), which are typically short label-like strings, and reference documents (REF) that serve as question or prompt text. To retain realistic source-side queries, extraction was restricted to selected curator-defined provenance sources, followed by normalization and deduplication within each CDE. Each query instance was linked to a stable CDE identifier. Additional extraction rules and provenance details are provided in the Supplementary Materials.

The caDSR-derived benchmark comprises 69,102 constructed query-CDE pairs, reduced to 68,659 after duplicate-pair removal. A query-level partition stratified by query source and provenance family gives the in-distribution train, Reranker Training, Validation and Test splits, totaling 64,346 pairs over 59,484 distinct queries. Three further caDSR-derived holdouts assess organizational and style shift and contribute 3,593 pairs: CCTG, OID ALT and CDASH. The independently assembled GDC and CIMAC evaluation sets contribute a further 203 pairs. Combining the in-distribution splits with all five holdouts, after filtering to queries whose gold CDE is present in the production catalog, gives an analysis-ready set of 68,142 pairs over 62,847 distinct queries. Benchmark construction used the January 2026 snapshot, whereas all fine-tuning and evaluation used a later production-oriented catalog of 62,976 CDE records. Exact split counts, catalog versions and filtering rules are reported in Supplementary Section S1.

### 2.2 Query and text representations

We evaluated multiple text representations for source queries and target CDEs. ALT and REF denote coarse query styles: ALT queries are short labels or identifiers, whereas REF queries are question- or prompt-like text. In the caDSR-derived datasets, these styles generally correspond to alternate-name and reference-document metadata, respectively; in independently assembled datasets, they were assigned by analogous query form.

For example, the ALT-style query CTP_CPS_prd_collect_dt_kn maps to *Product Collection Date Known Unknown Indicator*, whereas the REF-style query “Did patient require treatment with insulin therapy” maps to *Diabetes Mellitus Current Therapeutic Procedure Ind-2*, whose preferred question text is “Is patient being treated for diabetes.”

Query representations used these source-like strings, sometimes with concise permissible-value information. CDE representations combined metadata fields such as short name, long name, preferred definition, preferred question text, and permissible values, ranging from compact lexical forms to richer semantic descriptions.

Because permissible values can help distinguish enumerated elements, we also evaluated representations that included concise permissible-value summaries. True source-side permissible values were unavailable in the benchmark, so the query-side summary was a deliberately reduced, source-like rendering of the matched CDE’s permissible values, included only under predefined safeguards designed to limit leakage, whereas the CDE-side summary was a fuller rendering of the candidate CDE’s permissible values. Final representations were selected on the validation set; full recipes and construction rules are provided in the Supplementary Materials.

### 2.3 Models, baselines, and fine-tuning setup

This study evaluates three bi-encoders for source-to-CDE retrieval: the general-domain sentence-transformers/all-mpnet-base-v2 model (all-MPNet), the biomedical kamalkraj/BioSimCSE-BioLinkBERT-BASE model (BioSimCSE), and NeuML/pubmedbert-base-embeddings (PubMedBERT). Four comparators are reported. The official NCI CDE Match service is the production system described in the Introduction. For this study, official-service results are reported only for GDC and CIMAC because the other four evaluation sets were constructed from caDSR metadata fields searched directly by the live service, which could not be subjected to our query-level exact-match masking. The Python approximation to NCI CDE Match reimplements that service from its supplied PL/SQL source over the frozen production catalog, and unlike the live service it exposes its per-rule match types and can therefore carry the exact-match allowance described below. CDE Match-Fuzzy is our own CDE Match-inspired keyword retriever, which extends that matching flow with character- and word-level fuzzy and permissible-value evidence and supplies candidates to the final reranker. BM25 is a lexical baseline with no learned parameters; its weighting constants were left at standard values and only its query and CDE text representation was chosen on Validation, so it is deterministic and was run once. All retrieval methods used the same canonical query text and CDE catalog whenever their interfaces permitted it.

For semantic retrieval, query and CDE texts were encoded separately and ranked by cosine similarity. Each bi-encoder was evaluated in three stages: off-the-shelf inference, Phase 1 supervised contrastive fine-tuning with Multiple Negatives Ranking Loss (MNRL) or a symmetric bidirectional variant [21, 22], and Phase 2 refinement using mined hard negatives. Off-the-shelf screening compared query and CDE text representations, and the text representation and training objective carried forward into fine-tuning were selected separately for each model rather than shared across models. Fine-tuning used a standardized maximum sequence length for all models; Phase 1 used mixed-precision fine-tuning and Phase 2 was trained in FP32. All hyperparameters were selected on Validation, comparing configurations by the mean across two random seeds and prioritizing Recall@5, then Recall@10, and then MRR@100. A single seed was then retained from the winning seed-averaged configuration and used downstream. The internal test set and the distribution-shifted holdout sets were never used for tuning or model selection; they were evaluated only after validation-based selection was complete. Full training grids, loss-function screening, hard-negative strategies, and implementation details are provided in the Supplementary Materials.

FT-MPNet—all-MPNet [23] after the two-stage fine-tuning procedure described above—was selected as the final bi-encoder for downstream candidate generation and reranking. For each query, candidates retrieved by FT-MPNet were combined with candidates from the keyword retriever, and the union was deduplicated by CDE identifier to form the candidate pool passed to reranking. Bi-encoder, cross-encoder, and keyword features computed over this pool were supplied to a gradient-boosted reranker, which produced the final ranking.

Keyword retrieval used CDE Match-Fuzzy, an independently implemented, CDE Match-inspired method that combines exact, character- and word-level fuzzy, token-overlap and permissible-value evidence across five CDE metadata fields and contributes its top 10 candidates. It was selected over the Python approximation to NCI CDE Match on Validation. To limit circular exact lookup in the four caDSR-derived evaluation sets, both lexical methods used the same deterministic 70% query-level exact-match allowance; independently supplied GDC and CIMAC used 100%. Scoring and candidate-depth details are provided in Supplementary Section S5.2, and masking and sensitivity details are provided in Section S6.2.

Cross-encoder scoring evaluates each pooled query-candidate pair jointly. MiniLM [24], MedCPT and BGE [25] were compared off the shelf and after fine-tuning, and FT-MedCPT was selected by Validation Recall@5. Input representations, negative construction, sequence length and training details are provided in Supplementary Section S5.4.

## 3 Results

### 3.1 Descriptive dataset statistics

Table 1 summarizes the in-distribution splits and five holdouts. CCTG represents organizational REF-text shift, OID ALT compact identifier-style queries, CDASH standardized question text, GDC curated external metadata and CIMAC independently produced clinical-trial metadata.

### 3.2 Text-representation heatmap screening

We screened 4 × 10 query-CDE representation grids for all-MPNet, BioSimCSE and PubMedBERT on Validation without fine-tuning. Because the representation preferred by off-the-shelf models may not remain optimal after fine-tuning, we carried forward two pairs for each model: a prespecified information-rich anchor that had performed well in early fine-tuning experiments, and the best-performing non-anchor pair from the off-the-shelf screen. These were compared directly in the subsequent loss-function screening. Figure 2 reports all-MPNet, for which the anchor ranked second and raw query text against preferred question text ranked first; biomedical-model heatmaps are in the Supplement.

### 3.3 Representation and loss-function screening

Because fine-tuning can change how effectively a bi-encoder uses a given text representation, we did not assume that the best off-the-shelf representation would remain best after training. For each bi-encoder, we therefore compared the prespecified anchor and the best off-the-shelf representation under Multiple Negatives Ranking Loss (MNRL) [21] and a symmetric bidirectional contrastive variant [22] before running the main hyperparameter sweep. Both loss variants were evaluated over two seeds. Tables 2 and 3 report the all-MPNet screen and the selected representation-loss combination for each model; the biomedical-model screens appear in Figure S3.

### 3.4 Bi-encoder selection on Validation

After selecting a representation and loss for each bi-encoder, we compared the models after the full two-stage fine-tuning procedure. Phase 2 improved all three bi-encoders (Figure 3). All three fine-tuned models achieved similar Validation performance, indicating that task-specific fine-tuning was effective across different embedding backbones. all-MPNet achieved the highest mean Validation Recall@5 (0.9014 +/− 0.0008), followed closely by PubMedBERT (0.8966) and BioSimCSE (0.8941), and was selected as FT-MPNet. The margins were small relative to two-seed variability and no paired statistical test was performed, so we do not claim statistical superiority.

Supplementary Section S4.5 summarizes performance across off-the-shelf, Phase 1, and Phase 2 stages for the three bi-encoders.

### 3.5 Cross-encoder selection on Validation

Three cross-encoder backbones were compared using Validation Recall@5: ncbi/MedCPT-Cross-Encoder, BAAI/bge-reranker-base, and cross-encoder/ms-marco-MiniLM-L-6-v2 (Figure 4). FT-MedCPT was selected for the final reranker. All three backbones used the prespecified anchor representation from the bi-encoder experiments, pairing the raw query text and its permissible-value summary with the SN + DEC + DEF + PQT + PV CDE recipe, and no separate text-representation search was performed for MedCPT, BGE or MiniLM.

### 3.6 Keyword-algorithm selection and performance comparison

CDE Match-Fuzzy achieved Validation Recall@5 of 0.782 versus 0.728 for the Python approximation and was selected as the keyword retriever. Figure 5 reports full-set performance and performance among queries without available exact-match evidence for the gold CDE under the applicable allowance policy.

For this analysis, an exact lexical match means that the normalized query text is identical to at least one exact-match-eligible string for the gold CDE: its long name, a question-text string from its reference documents (including preferred or alternate question text) or an alternate name. Because the four caDSR-derived evaluation sets were constructed from metadata linked to their gold CDEs, unrestricted exact lookup could reveal the answer for many queries; exact matching to the gold CDE was therefore permitted for a deterministic 70% of queries. GDC and CIMAC were assembled independently and used a 100% allowance; the non-exact subset comprises queries for which no exact lexical match to the gold CDE was available under the applicable allowance policy.

### 3.7 End-to-end algorithm

The selected system combines FT-MPNet top-20 and CDE Match-Fuzzy top-10 candidates, deduplicates them by CDE public identifier, scores every pair with FT-MedCPT, and applies the HGBC reranker (Figure 6).

### 3.8 Final system performance

The relative strengths of semantic and keyword retrieval varied across datasets, while the final hybrid reranker remained strong across these shifts. Together with the increased candidate coverage from combining the two retrievers (Table S5), this supports the value of integrating lexical and semantic evidence rather than relying on a single retrieval method.

At Stage 1, combining FT-MPNet and CDE Match-Fuzzy increased gold-CDE coverage over either retriever alone on every dataset (Table S5). A fixed-candidate-pool ablation further showed that lexical/keyword-oriented evidence contributed most to final ranking, suggesting that detailed lexical cues are especially useful for distinguishing among already-plausible CDE candidates (Figure S6).

The official NCI CDE Match service was evaluated only on GDC and CIMAC because it directly queries the caDSR alternate-name and question-text fields used to construct Test, CCTG, OID ALT, and CDASH. Evaluation on those four datasets would therefore be circular, and the live service could not be subjected to the query-level masking used in our controlled experiments. For CDE Match-Fuzzy and the Python approximation, exact matching to the gold CDE was permitted for a deterministic 70% of queries on the four caDSR-derived datasets and 100% on the independently assembled GDC and CIMAC sets. On GDC and CIMAC, both the official service and the Python approximation achieved Recall@5 of 0.986 and 0.679, respectively.

On GDC, the official service and Python approximation each retrieved the gold CDE for 71 of 72 queries, compared with 70 of 72 for the final reranker; the methods failed on different queries. On CIMAC, removing the 18 of 131 queries that recurred in training with the same gold CDE reduced Recall@5 from 0.802 to 0.779 for the final reranker and from 0.679 to 0.637 for the Python approximation, without changing method order (Table S6).

Exact-match allowance sensitivity analyses further showed that the final HGBC was comparatively robust to lexical exact-match availability (Figures S7 and S8). The fixed HGBC trained at the 70% allowance varied substantially less than either lexical method across the 0–100% allowance range.

### 3.9 Limitations

This study has several limitations. all-MPNet was selected on Validation, and the Phase 2 comparison rests on two seeds per configuration; the margins between the three fine-tuned bi-encoders are small (0.0048 Recall@5 over PubMedBERT and 0.0073 over BioSimCSE) and no paired statistical test was performed, so we make no claim that all-MPNet is significantly better than the biomedical models. On the six evaluation datasets the three fine-tuned bi-encoders are similarly close (Table S4), and the external evaluations are best read as evidence of robustness under distribution shift rather than as a ranking of model quality. The external sets are also not fully independent of training data under a text-plus-gold criterion (Table S6), and cross-encoder results come from a single training run per backbone, so they carry no seed variance. Finally, we report Recall@5 and related top-K measures; precision was not evaluated.

## 4 Discussion and Conclusion

Source-to-CDE mapping benefits from complementary evidence. Lexical matching resolves literal identifiers, abbreviations and short schema-like labels, where one rare token is decisive; dense semantic retrieval resolves the complementary case, in which a source element and its target CDE express the same concept in different words. Because the two families fail in different places, combining them is productive rather than redundant: on OID ALT, CDE Match-Fuzzy achieved Recall@5 of 0.702 versus 0.428 for FT-MPNet, whereas on the internal Test set FT-MPNet achieved 0.912 versus 0.773 for CDE Match-Fuzzy.

The later stages exploit that complementarity. The cross-encoder scores each query–candidate pair jointly, allowing it to model fine-grained interactions between specific query and CDE terms that independent bi-encoder embeddings may not fully preserve. The reranker then combines these interaction scores with lexical and semantic retrieval signals. When exact-match availability was varied from 0% to 100%, the HGBC trained at the 70% allowance maintained more stable Recall@5 than the lexical methods. Most of the remaining change in its performance arose because varying exact-match availability also changed whether the correct CDE entered the candidate pool.

Across the six evaluation datasets the integrated system achieved the highest Recall@5 among the evaluated methods on five. On GDC, near ceiling for lexical methods, direct CDE Match lookup retrieved one additional query of 72 and the systems failed on different queries; on CIMAC the integrated system was substantially stronger than the official service. These results indicate robust performance across heterogeneous datasets. This robustness matters because the final ranking is not tightly coupled to a particular level of exact-match availability. Overall, robust source-to-CDE mapping appears to depend not on any single retrieval signal, but on integrating complementary evidence within a unified ranking framework.

## Supplementary Material

1

## Figures and Tables

**Figure 1:**
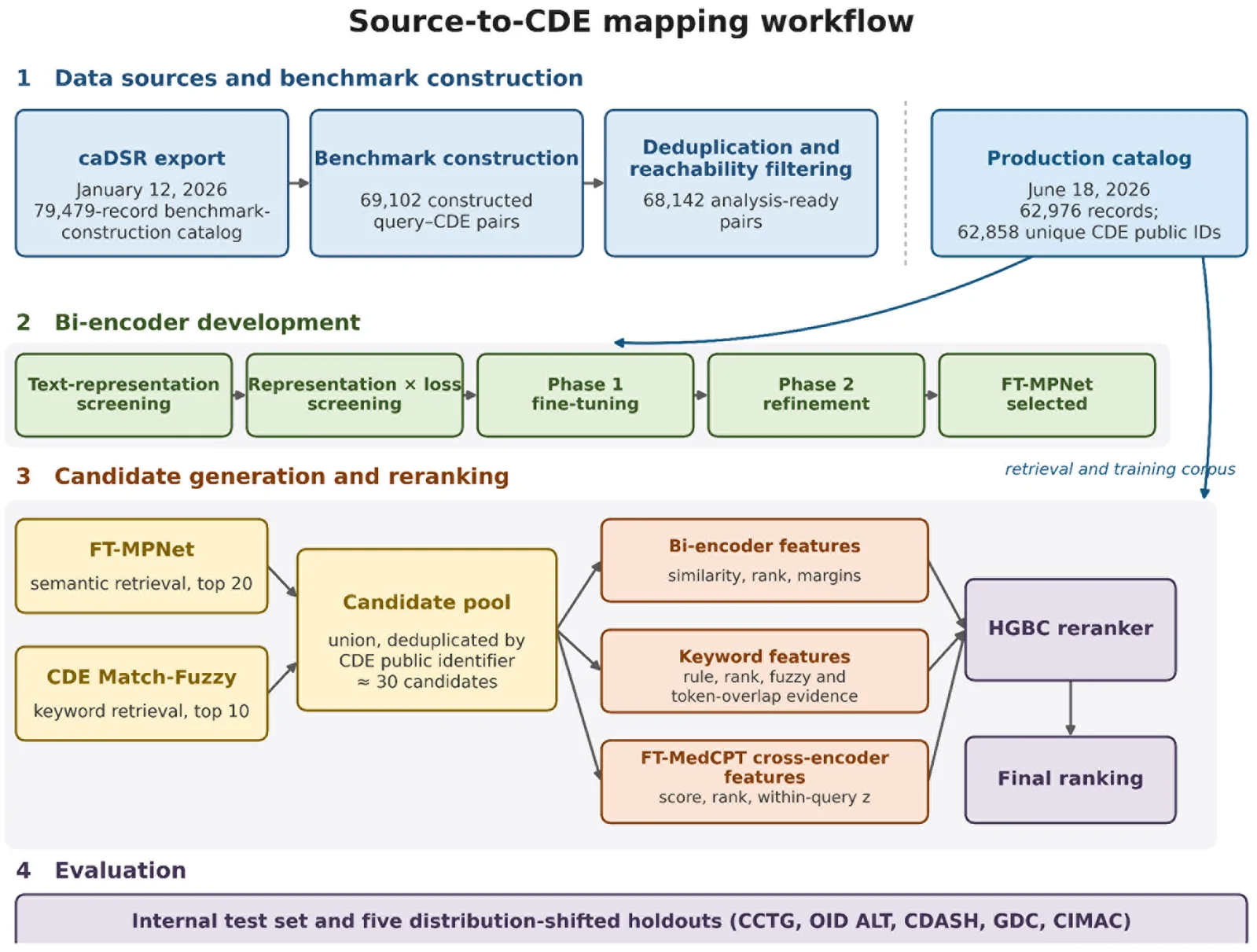
Source-to-CDE workflow. caDSR metadata yields benchmark queries, gold links, and splits; training and retrieval use a later production catalog. Bi-encoders are screened and fine-tuned, and FT-MPNet and CDE Match-Fuzzy form a candidate pool. Bi-encoder, keyword, and FT-MedCPT cross-encoder features are computed for each pooled candidate and supplied to the HGBC, which produces the final ranking. Evaluation uses the internal test set and five distribution-shifted holdouts.

**Figure 2:**
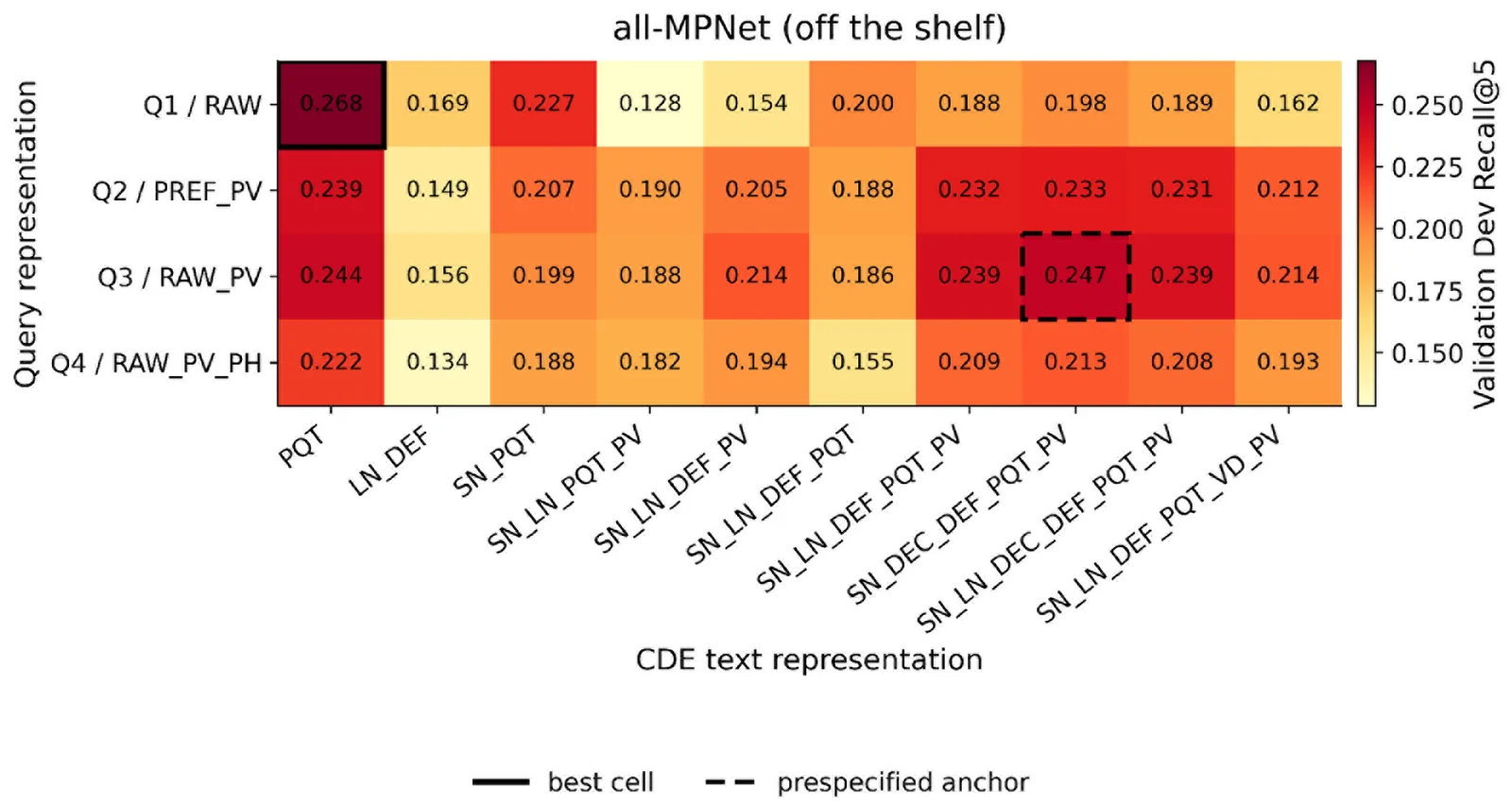
Off-the-shelf all-MPNet Validation Recall@5 across the 4 x 10 representation grid (n = 4,234 query–CDE pairs from 3,934 distinct Validation queries). The solid box marks the best cell and the dashed box the prespecified anchor.

**Figure 3:**
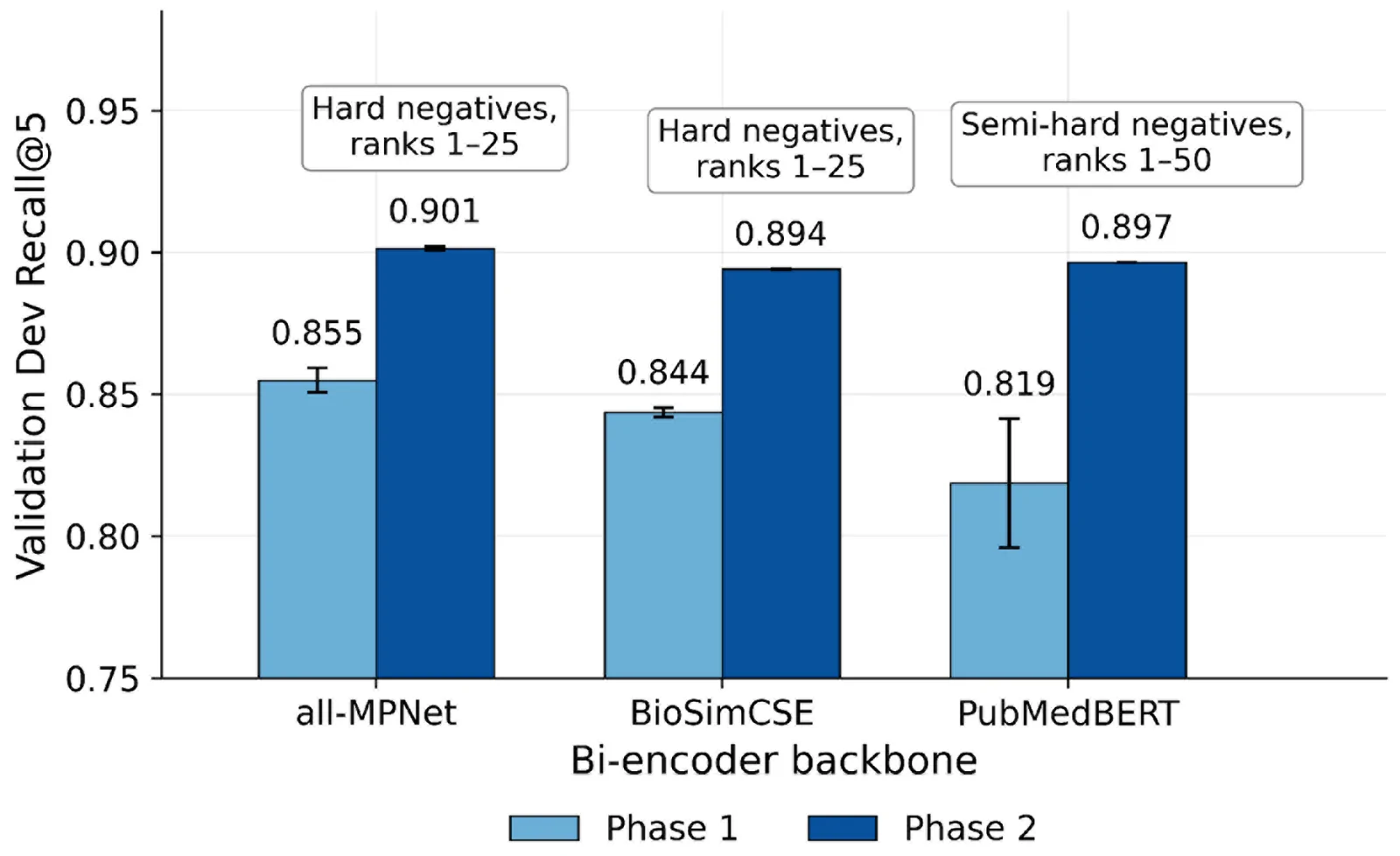
Validation Recall@5 after Phase 1 and Phase 2 fine-tuning (two-seed mean and sample SD). Selected Phase 2 strategies used hard negatives sampled from ranks 1–25 for all-MPNet and BioSimCSE and semi-hard negatives sampled from ranks 1–50 for PubMedBERT. Recall is computed over n = 4,234 query–CDE pairs from 3,934 distinct Validation queries.

**Figure 4:**
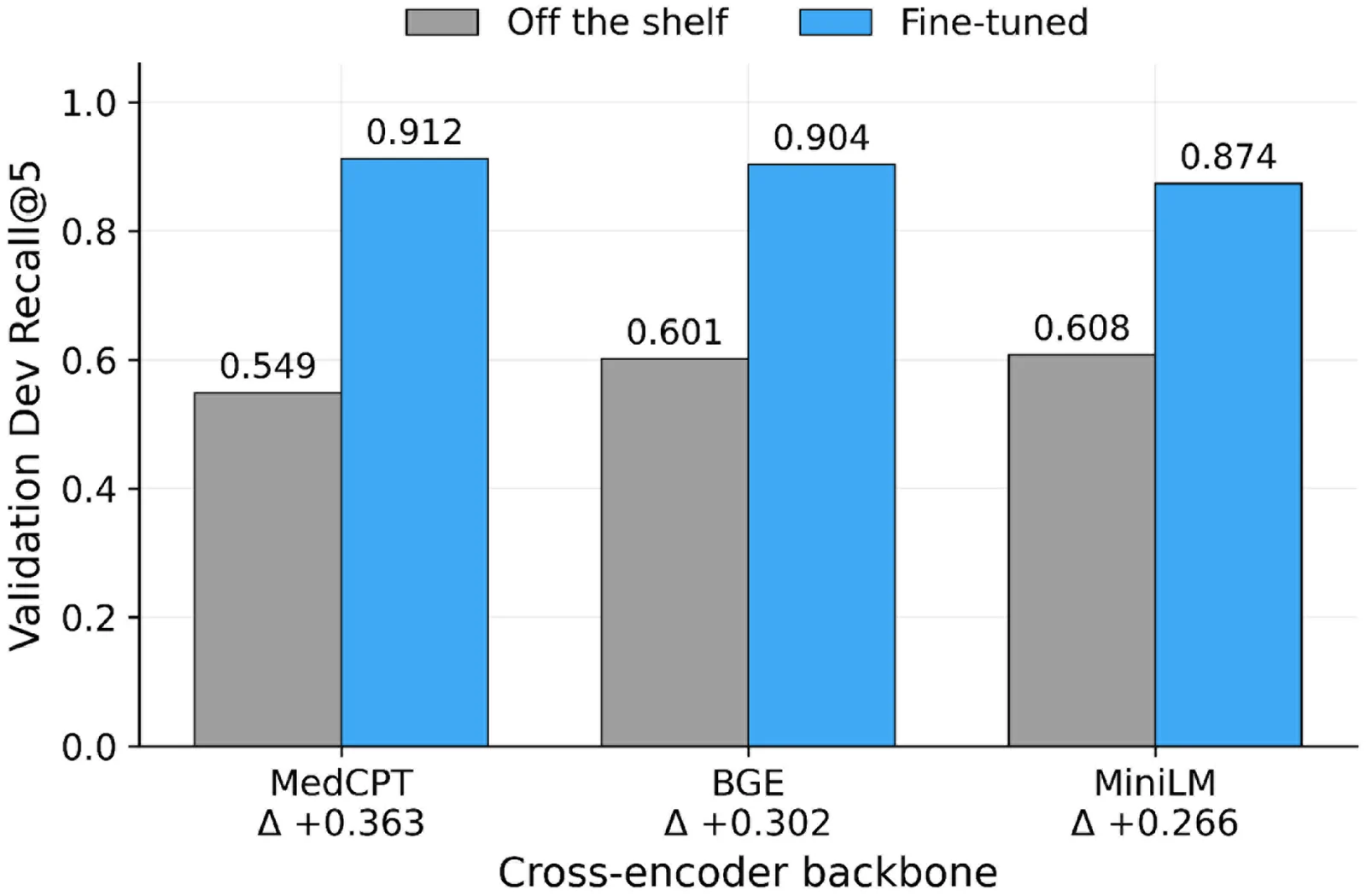
Off-the-shelf and fine-tuned cross-encoder Validation Recall@5. Δ denotes the difference between fine-tuned and off-the-shelf Recall@5. Each backbone used one fine-tuning run; FT-MedCPT was selected.

**Figure 5:**
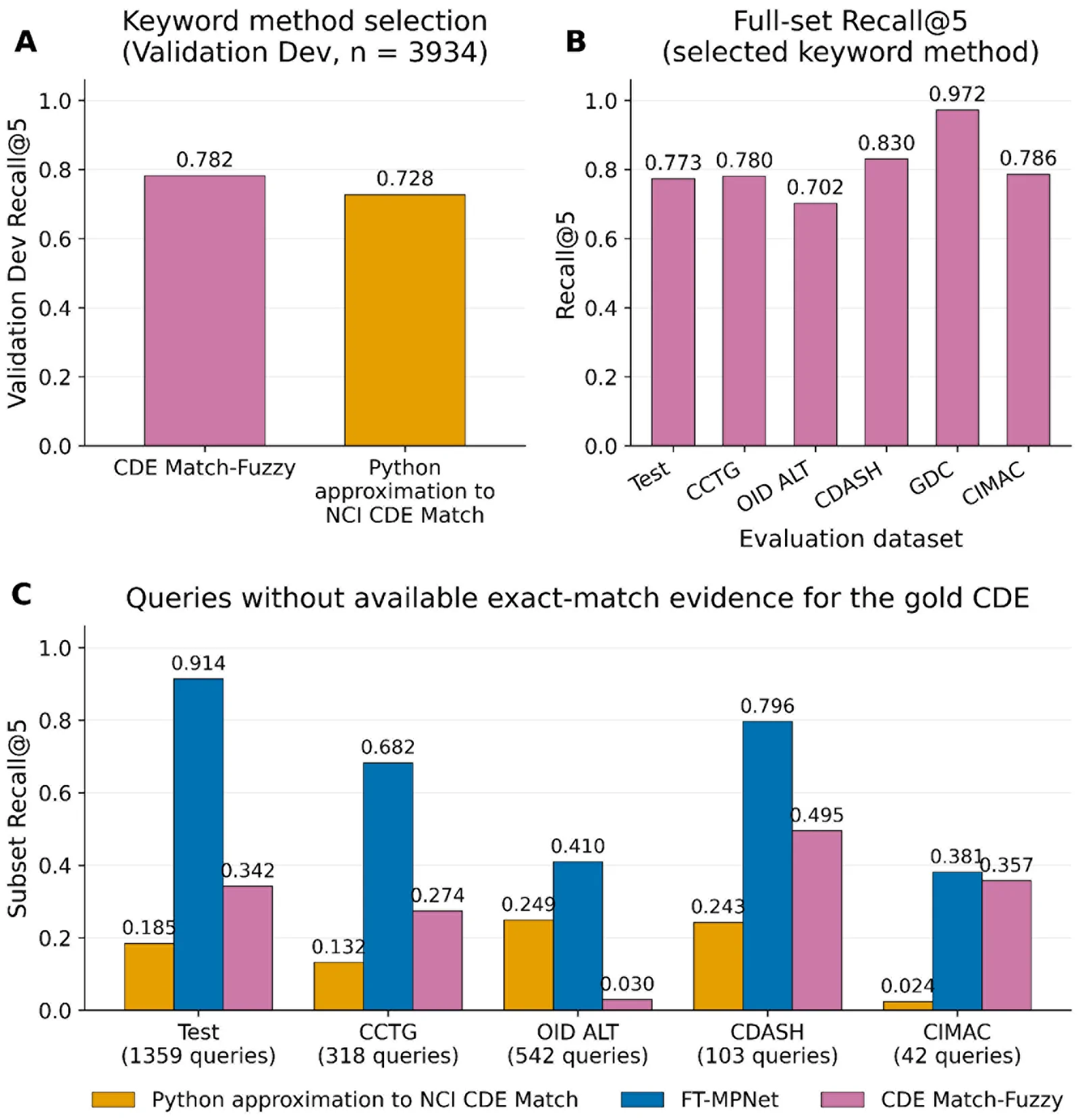
Keyword-method selection and evaluation: (A) Validation comparison; (B) CDE Match-Fuzzy Recall@5 across evaluation sets; and (C) Recall@5 among queries without available exact-match evidence for the gold CDE under the applicable allowance policy. GDC is omitted from panel C because only two such queries remain.

**Figure 6:**
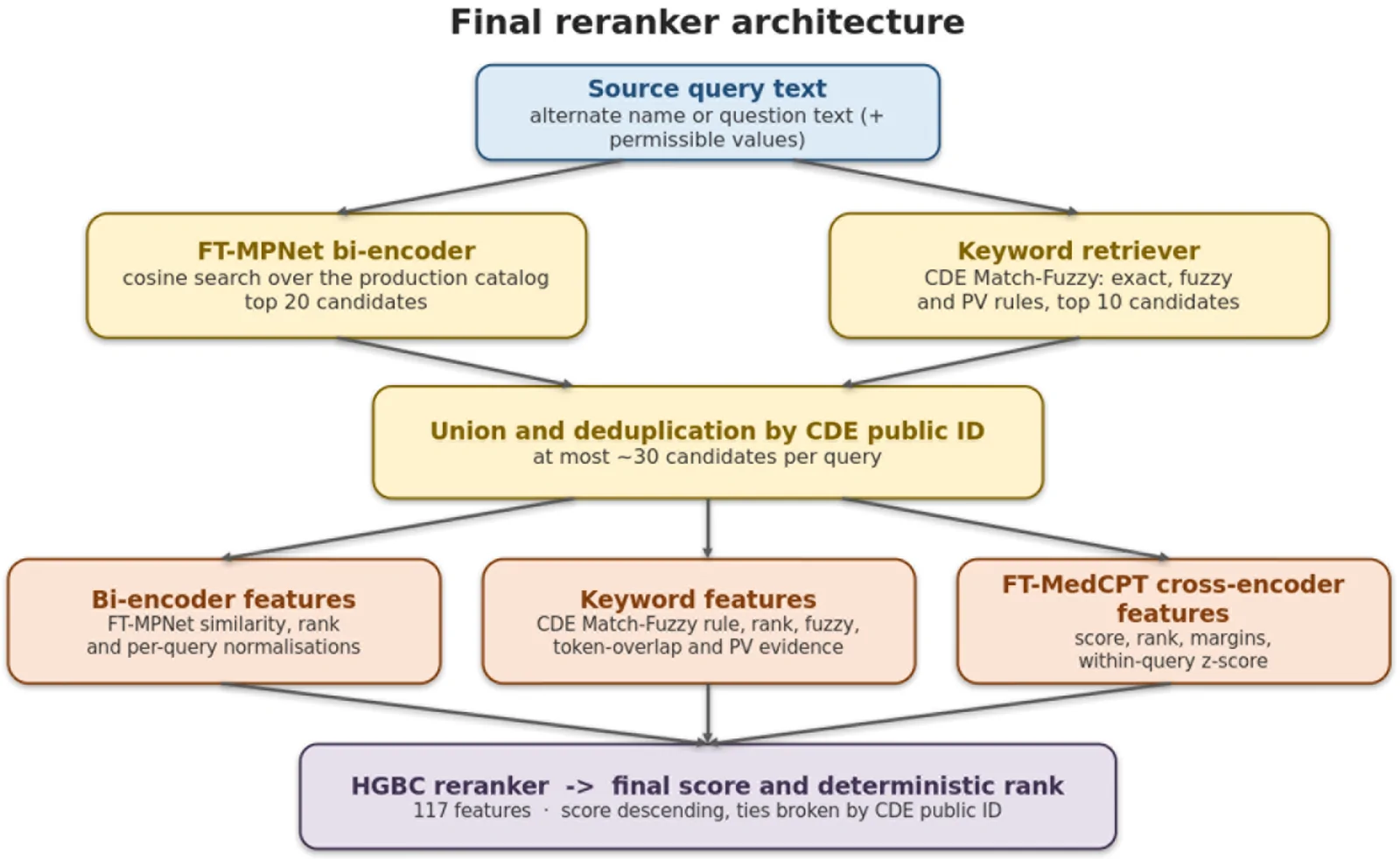
End-to-end reranker. FT-MPNet and CDE Match-Fuzzy form an approximately 30-candidate pool. For each candidate, the system computes bi-encoder, keyword, and FT-MedCPT cross-encoder features; the HGBC combines 117 lexical and semantic features to produce the final ranking. Ties are broken by CDE public identifier.

**Table 1.** Dataset composition and evaluation-set roles. Query counts are reported after canonical deduplication and reachable-gold filtering. The ALT/REF mixture gives the share of ALT-style versus REF-style queries, as defined in Section 2.2.

Dataset	Queries	ALT/REF mix	Shift summary
Train	47,645	ALT 40.5% / REF 59.5%	In-distribution caDSR pool (80% of query_ids); not filtered for whether the gold CDE is present in the production catalog
Reranker Training	3,946	ALT 40.6% / REF 59.4%	In-distribution; in-sample reference split (not used for model selection)
Validation	3,934	ALT 40.6% / REF 59.4%	In-distribution; primary model-selection split
Test	3,959	ALT 40.7% / REF 59.3%	same ALT/REF mixture as Train
CCTG	1,097	ALT 0.0% / REF 100.0%	Organizational shift: all REF-style question text from a held-out CCTG context
OID ALT	1,766	ALT 100.0% / REF 0.0%	Naming-style shift: all ALT-style short identifier-like queries
CDASH	324	ALT 0.0% / REF 100.0%	Standards-vocabulary shift: REF-style CDISC CDASH question text
GDC	72	ALT 45.8% / REF 54.2%	Curated external repository shift: GDC preferred-question-text + alternate-name queries
CIMAC	131	ALT 100.0% / REF 0.0%	Real-world clinical-trial submission shift: ALT-style schema-like data-element requests with PVs

**Table 2.** all-MPNet representation-by-loss screen on Validation. Each cell is the mean of two seeds with the sample standard deviation; the selected condition is marked. The anchor pair is raw query + PV summary x SN + DEC + DEF + PQT + PV and the best pair is the highest-scoring non-anchor pair from Section 3.2.

Representation pair	Query	CDE recipe	Loss	Val Recall@5 (mean)	SD
anchor	raw query + PV summary	SN + DEC + DEF + PQT + PV	MNRL	0.8368	0.0023
anchor	raw query + PV summary	SN + DEC + DEF + PQT + PV	Symmetric bidirectional	0.8392 (selected)	0.0013
best	raw query	PQT	MNRL	0.6436	0.0040
best	raw query	PQT	Symmetric bidirectional	0.6503	0.0042

**Table 3.** Selected query representation, CDE representation and loss for each retained bi-encoder, with the two-seed mean Validation Recall@5 and sample standard deviation from the representation-by-loss screen.

Model	Query representation	CDE representation	Loss	Val Recall@5 (mean)	SD
all-MPNet	raw query + PV summary	SN + DEC + DEF + PQT + PV	Symmetric bidirectional	0.8392	0.0013
BioSimCSE	prefixed query + PV summary	SN + LN + PQT + PV	Symmetric bidirectional	0.8344	0.0033
PubMedBERT	raw query + PV summary	SN + DEC + DEF + PQT + PV	MNRL	0.8227	0.0175

**Table 4:** summarizes Recall@5 for the final reranker and evaluated benchmarks. Table 4. Recall@5 for the final reranker and evaluated benchmarks. FT-MedCPT reranks the same approximately 30-candidate pool used by the HGBC rather than retrieving from the full catalog. All tunable methods were selected on Validation.

Dataset	Final reranker	Python approximation to NCI CDE Match	BM25	FT-MPNet	CDE Match-Fuzzy	FT-MedCPT (top-30 pool reranking)
Test	**0.971**	0.719	0.361	0.912	0.773	0.914
CCTG	**0.909**	0.739	0.392	0.705	0.780	0.718
OID ALT	**0.832**	0.770	0.176	0.428	0.702	0.564
CDASH	**0.920**	0.750	0.710	0.840	0.830	0.864
GDC	0.972	**0.986**	0.542	0.833	0.972	0.903
CIMAC	**0.802**	0.679	0.160	0.565	0.786	0.595

## Data Availability

Source code, reproducibility materials, and the processed GDC and CIMAC evaluation datasets used in this study are publicly available in the DEMap repository at https://github.com/jhtanis/DEMap. The CIMAC evaluation set was derived from the CIMAC-CIDC clinical data-element template, available from the National Cancer Institute at https://dctd.cancer.gov/research/networks/precision-medicine-oncology/cimac-cidc. The caDSR-derived benchmark and production catalog were constructed from the January 12, 2026 and June 18, 2026 caDSR CDE XML exports, respectively; these dated exports are available from the caDSR CDE XML Archive at https://cadsr.nci.nih.gov/ftp/caDSR_Downloads/CDE/XML_Archive/. Additional provenance and dataset-construction details are provided in the repository documentation and Supplementary Materials.
