# Supplementary material for: Hybrid Semantic–Lexical Retrieval for Source-to-CDE Mapping in the NCI caDSR
Source: bioRxiv. 2026 Oct 20:2026.10.14.758967. Preprint. [Version 1] doi: 10.64898/2026.10.14.758967 (PMC13738425; doi:10.64898/2026.10.14.758967)
Supplement: Supplementary file 1 [file NIHPP2026.10.14.758967v1-supplement-1.pdf]

bioRxiv preprint doi: https://doi.org/10.64898/2026.10.14.758967; this version posted October 20, 2026. The copyright holder for this preprint (which was not certified by peer review) is the author/funder, who has granted bioRxiv a license to display the preprint in perpetuity. It is made available under a CC-BY 4.0 International license.

Supplementary Materials

S1. Data sources, benchmark construction, and evaluation sets

S1.1 Data sources and retrieval task

This study uses three data resources: a caDSR-derived benchmark and three caDSR-derived holdouts constructed from the January 12, 2026 XML export; independently assembled GDC and CIMAC evaluation sets; and a production-oriented CDE catalog derived from the June 18, 2026 caDSR export. The January export supplied the source-like queries and gold CDE links used for benchmark construction, whereas the June catalog supplied the candidate CDEs used for training and retrieval.

The task is to map a source-like query string to its accepted caDSR Common Data Element (CDE). Retrieval methods rank candidate CDEs from the production catalog, and a query is counted as successfully retrieved when an accepted gold CDE appears within the evaluated rank cutoff.

S1.2 Query construction and provenance

The January export was parsed into normalized tables containing CDE metadata, alternate names, reference documents, and permissible values. Queries were derived from alternate names (ALT), which are typically short labels or identifier-style strings, and reference documents (REF), which are typically questions or prompt text.

Extraction was restricted to curator-defined provenance sources. ALT queries were selected using specified combinations of alternate-name type and context, together with deterministic concatenation rules where applicable. REF queries were selected from question-like document types, including Preferred Question Text, Alternate Question Text, and Application Standard Question Text. Queries were normalized, deduplicated within each CDE and provenance group, and linked to stable CDE public identifiers.

ALT and REF query styles are defined in Section 2.2. For the caDSR-derived datasets, these styles correspond to queries harvested from alternate-name and reference-document metadata, respectively. Provenance family is a separate curator-harvest grouping used for stratification and holdout construction; a provenance family can contain both ALT- and REF-style queries.

S1.3 Benchmark splits and evaluation sets

The benchmark contains 69,102 constructed query–CDE pairs. After canonical deduplication and filtering to queries whose gold CDE was present in the production catalog, 68,142 pairs remained, representing 62,847 distinct queries.

The in-distribution data comprised 64,346 pairs representing 59,484 distinct queries. These were partitioned at the query level into Train, Reranker Training, Validation, and Test, with stratification by query source and provenance family. Approximately 80% of the queries were assigned to Train, with the remainder divided among the three evaluation splits.

Five additional holdouts were used to evaluate distribution shift. CCTG, OID ALT, and CDASH were derived from the January caDSR export but separated by provenance or query style. GDC and CIMAC were assembled independently of that export and used only for external evaluation. Final dataset sizes are reported in Table 1.

S1.4 CDE catalogs and reachability filtering

Two caDSR catalogs served different purposes. The January benchmark-construction catalog contains 79,479 records representing 78,533 unique CDE public identifiers. It was not restricted to production-eligible records and included 15,852 records with a retired workflow status, together with a small number of records from administrative contexts. This catalog defined the source metadata and gold CDE links used to construct the caDSR-derived queries.

The June production catalog contains 62,976 records representing 62,858 unique CDE public identifiers. It was created from a 79,827-record export by removing 16,846 retired records and 21 records from administrative TEST or Training contexts; 16 records met both exclusion criteria. This filtering was administrative and unrelated to the machine-learning data splits.

The production catalog supplied CDE text for training pairs, in-batch negatives, candidate generation, and evaluation. Evaluation sets were filtered to retain only queries whose accepted gold CDE was present in this catalog.

bioRxiv preprint doi: https://doi.org/10.64898/2026.10.14.758967; this version posted October 20, 2026. The copyright holder for this preprint (which was not certified by peer review) is the author/funder, who has granted bioRxiv a license to display the preprint in perpetuity. It is made available under a CC-BY 4.0 International license.

Because the catalog was filtered and derived from a later export, 622 training pairs lacked target CDE text and were removed, leaving 50,972 training pairs.

S1.5 Training–evaluation overlap

We assessed whether source-to-CDE mappings in the five holdouts duplicated mappings in Train. We first compared query identifiers. CCTG, OID ALT, GDC, and CIMAC shared no query identifiers with Train. CDASH shared 14 identifiers with the in-distribution data, but the associated accepted gold CDE sets were disjoint; these records therefore did not represent duplicate mappings and were retained.

We then applied a stricter, content-based definition: identical normalized query text paired with the same gold CDE public identifier. Under this definition, repeated mappings occurred in 8 of 1,097 CCTG queries, 7 of 1,766 OID ALT queries, 14 of 324 CDASH queries, 4 of 72 GDC queries, and 18 of 131 CIMAC queries. Table S6 reports sensitivity analyses after removing these repeated mappings, and leakage-filtered evaluation files are provided alongside the original sets.

S2. Dataset characterization

S2.1 ALT/REF style and provenance composition

Table S1. ALT/REF-style and provenance composition of the training and evaluation datasets.

Dataset	Queries	ALT-style share	Mean query tokens	Code-like queries	Top family	Top family share
Train	47,645	40.5%	9.8	39.7%	NMDP	34.1%
Validation	3,934	40.6%	10.0	39.7%	NMDP	34.2%
Test	3,959	40.7%	9.9	39.9%	NMDP	34.2%
CCTG	1,097	0.0%	10.2	1.5%	CCTG	100.0%
OID ALT	1,766	100.0%	3.4	98.7%	OID	100.0%
CDASH	324	0.0%	3.2	6.8%	CDASH	100.0%
GDC	72	45.8%	4.4	45.8%	GDC Question Text	54.2%
CIMAC	131	100%	2.8	100.0%	N/A	N/A

- **Queries:** number of queries after canonical deduplication and reachable-gold filtering.
- **ALT-style share:** share of unique queries classified as ALT style rather than REF style, using the definitions in Section 2.2.
- **Mean query tokens:** mean number of tokens per query, where a token is a maximal run of [A-Za-z0-9] characters in the bare source string (no ALT_NAME:/REF_TEXT: label and no permissible-value block).
- **Code-like queries:** share of queries that are a single schema-style identifier with no internal whitespace.
- **Top family:** most common provenance family in that dataset.
- **Top family share:** share of queries belonging to that provenance family.

Train, Validation, and Test were constructed to have similar ALT/REF-style and provenance profiles. The holdouts intentionally differ in style: CCTG and CDASH contain REF-style question text, OID ALT and CIMAC contain ALT-style identifier-like queries, and GDC combines both styles. The provenance-family columns separately describe the curator or external-source groupings represented in each dataset. For CIMAC, these fields are N/A because the evaluation set spans multiple clinical trials rather than coming from known provenance families.

S2.2 Query-style composition

Table S2. Query-style composition of the training and evaluation datasets.

bioRxiv preprint doi: https://doi.org/10.64898/2026.10.14.758967; this version posted October 20, 2026. The copyright holder for this preprint (which was not certified by peer review) is the author/funder, who has granted bioRxiv a license to display the preprint in perpetuity. It is made available under a CC-BY 4.0 International license.

Dataset	Dominant style	Mean query tokens	Code-like queries	Underscore-containing	Question marks	Token unigram JSD vs Train
Train	natural_language	9.8	39.7%	35.9%	30.6%	0.000
Validation	natural_language	10.0	39.7%	36.1%	31.3%	0.042
Test	natural_language	9.9	39.9%	35.8%	29.8%	0.043
CCTG	natural_language	10.2	1.5%	0.0%	32.6%	0.206
OID ALT	schema_like	3.4	98.7%	99.8%	0.0%	0.620
CDASH	natural_language	3.2	6.8%	0.0%	0.3%	0.495
GDC	mixed	4.4	45.8%	45.8%	1.4%	0.494
CIMAC	schema_like	2.8	100.0%	89.3%	0.0%	0.481

- **Dominant style:** coarse style label assigned to the dataset (natural_language, schema_like or mixed).
- **Mean query tokens:** mean number of tokens per query, a token being a maximal run of [A-Za-z0-9] characters in the bare source string.
- **Code-like queries:** share of queries that are a single schema-style identifier with no internal whitespace.
- **Underscore-containing:** share of queries containing at least one underscore.
- **Question marks:** share of queries containing at least one question mark.
- **Token unigram JSD vs Train:** Jensen-Shannon divergence (natural logarithm, additive smoothing 1e-8) between the dataset's token unigram distribution and the Train reference distribution; larger values indicate a stronger lexical shift away from training.

Higher token-distribution divergence indicates a stronger shift away from the training query distribution. OID ALT and CIMAC are schema-like: nearly every query is a single identifier, underscores are near-universal and question marks are absent. CDASH is short natural language rather than schema text. Divergence from Train increases with distance from the training distribution, in the order Validation 0.042 $<$ Test 0.043 $<$ CCTG 0.206 $<$ CIMAC 0.481 $<$ GDC 0.494 $<$ CDASH 0.495 $<$ OID ALT 0.620.

S3. Text-representation design and screening

S3.1 CDE metadata fields

CDE text representations were constructed from fields available in the caDSR XML export: preferred name (abbreviated here as short name, SN), long name (LN), preferred definition (DEF), data element concept long name (DEC), preferred question text (PQT), value domain (VD), and permissible-value summary (PV). SN is the closest lexical analogue of a source variable name, whereas LN, DEF, DEC, and PQT provide additional semantic context. PV summaries were included only for enumerated CDEs.

S3.2 Permissible-value summaries

When permissible values were available, we constructed PV summaries for the CDE side and, in selected cases, for the query side. Because true source-side permissible values were unavailable, both summaries were derived from the permissible-value inventory of the matched CDE. Query-side PV summaries were included only when the case was not judged to be leakage-prone.

To reduce leakage, query-side PV summaries were designed to be more conservative and more source-like than CDE-side summaries. The query side used a lower display cap, allowed mild preference for generic concepts such as yes/no, and favored code-oriented rendering when available. The CDE side used preferred meaning labels and a higher display cap, yielding a more formal representation. Additional safeguards omitted selected small PV sets on the query side, retried deterministic sampling when rendered query and CDE summaries were identical, and suppressed cases in which the query-side summary was a strict subset of the CDE-side summary.

bioRxiv preprint doi: https://doi.org/10.64898/2026.10.14.758967; this version posted October 20, 2026. The copyright holder for this preprint (which was not certified by peer review) is the author/funder, who has granted bioRxiv a license to display the preprint in perpetuity. It is made available under a CC-BY 4.0 International license.

S3.3 Query text variants

We evaluated four query representations:

- Q1: raw query text only
- Q2: prefixed query text, with PV summary when available
- Q3: raw query text plus PV summary when available
- Q4: raw query text plus PV summary when available, with an explicit missing-value block when PV summary was absent

Q1 is the simplest source-like representation. Q2 tests whether explicit labels for query content and answer-choice information improve retrieval. Q3 removes those labels to test whether the extra structure in Q2 is helpful. Q4 further tests whether explicitly marking missing PV information is more helpful than leaving it absent. Together, these variants assess whether answer-choice information and missingness cues improve source-to-CDE matching.

For independently assembled datasets, Q2 prefixes were assigned from the ALT/REF query-style classification rather than from caDSR metadata provenance.

S3.4 CDE text recipes

The ten CDE text recipes evaluated in the representation grid, named by the fields they concatenate, are: PQT; LN + DEF; SN + PQT; SN + LN + DEF + PQT; SN + LN + DEF + PV; SN + LN + PQT + PV; SN + LN + DEF + PQT + PV; SN + DEC + DEF + PQT + PV; SN + LN + DEF + PQT + VD + PV; SN + LN + DEC + DEF + PQT + PV. Fields are joined in the order listed with a " | " separator and a field label prefix.

SN values were omitted when they were purely numeric or version-like strings, since these were unlikely to aid semantic matching. For non-enumerated CDEs, the PV component was omitted.

S3.5 Permissible-value overlap analysis

Table S3. PV-summary overlap between query-side and CDE-side permissible values.

Group	Rows with query+CDE PV	Normalized block match	Query shorter than CDE	Mean Jaccard Overlap
overall	39,391	0.4%	30.1%	0.168
ENUM	24,543	0.4%	45.2%	0.153
BINARY_WITH_UNKNOWN	3,074	0.1%	0.0%	0.333
BINARY_WITH_NA	8,816	0.0%	0.0%	0.123

- **Group:** PV-type subgroup used for the overlap analysis, such as overall, ENUM, or binary-family types. Rows with query+CDE PV: number of rows where both the query and the matched CDE have PV summaries present.
- **Normalized block match:** percentage of rows where the full query-side PV block matches the CDE-side PV block after normalization.
- **Query shorter than CDE:** percentage of rows where the query-side PV summary contains fewer items than the CDE-side PV summary.
- **Mean Jaccard Overlap:** mean Jaccard overlap between the query-side and CDE-side permissible-value item sets.

Whole-block agreement between query-side and CDE-side permissible values is rare: over the 39,391 rows in which both sides carry a permissible-value summary, only 0.4% match as a normalized block, while the mean item-level Jaccard overlap is 0.168 and 30.1% of query-side summaries are shorter than the CDE-side summary. Query-side permissible values therefore contribute partial, not duplicated, evidence.

S3.6 Off-the-shelf representation screening

We screened the final 40 query-CDE representation combinations, formed from four query variants and ten CDE recipes, separately for all-MPNet, BioSimCSE, and PubMedBERT. For each model, the prespecified SN + DEC +

bioRxiv preprint doi: https://doi.org/10.64898/2026.10.14.758967; this version posted October 20, 2026. The copyright holder for this preprint (which was not certified by peer review) is the author/funder, who has granted bioRxiv a license to display the preprint in perpetuity. It is made available under a CC-BY 4.0 International license.

DEF + PQT + PV anchor and the best-performing non-anchor representation pair on Validation were carried forward to the 2 x 2 representation and loss-function screen. This screening stage used off-the-shelf inference at each model's own effective maximum sequence length: 384 tokens for all-MPNet, 256 tokens for BioSimCSE (whose 128-token checkpoint value is raised to the model loader's 256-token floor), and 512 tokens for PubMedBERT. Fine-tuning subsequently standardized the maximum sequence length across all models, so these off-the-shelf values apply only to this stage.

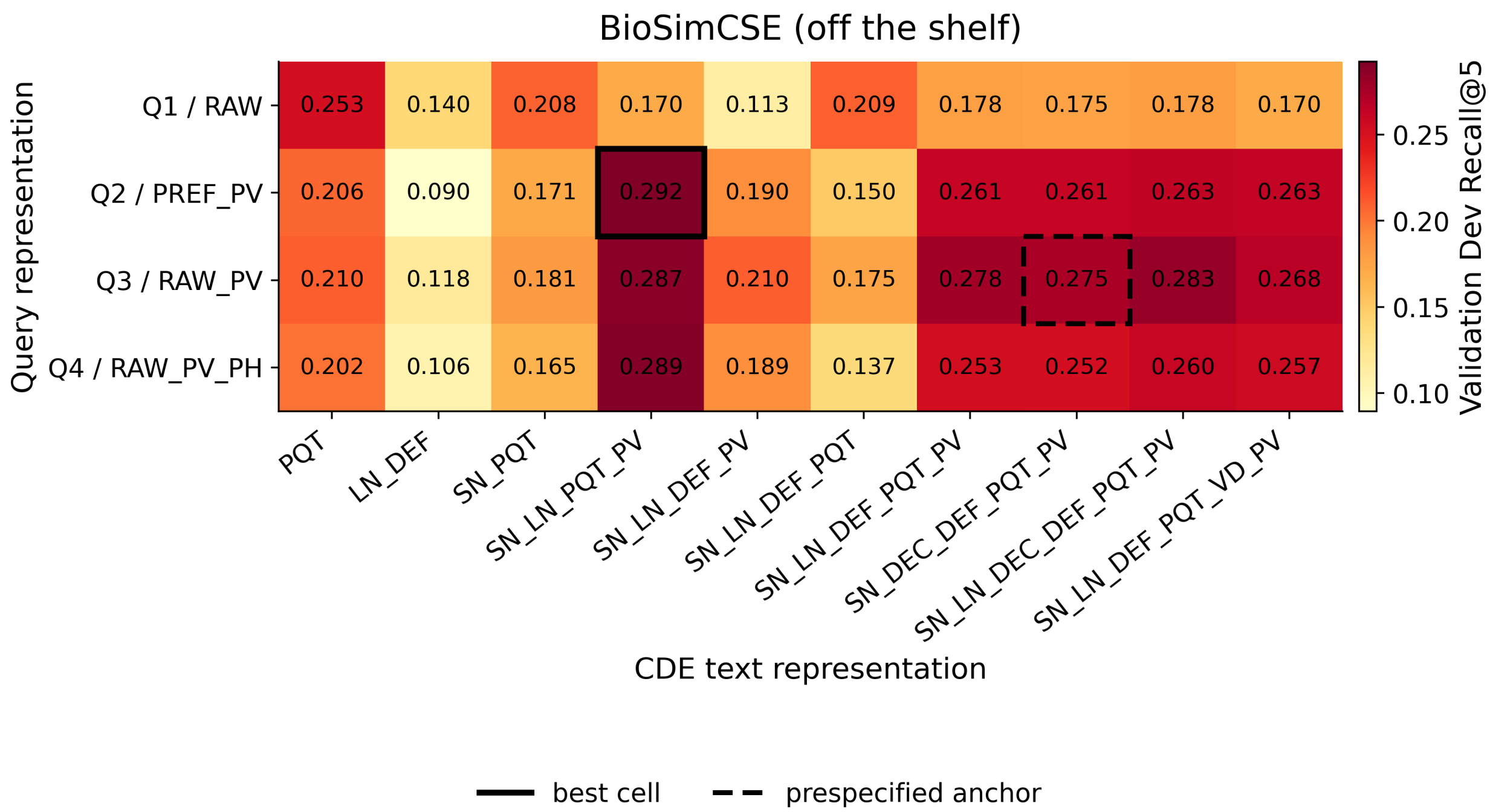

Figure S1: Validation Recall@5 across the 4 x 10 query-CDE text-representation grid for off-the-shelf BioSimCSE (n = 4,234 query–CDE pairs from 3,934 distinct Validation queries). The solid box marks the best cell and the dashed box the prespecified anchor.

S4. Bi-encoder development and evaluation

S4.1 Bi-encoder models, retrieval setup, and selection procedure

We evaluated three bi-encoders. The general-domain model was sentence-transformers/all-mpnet-base-v2 (all-MPNet), a general-purpose bi-encoder based on MPNet.

The biomedical models were kamalkraj/BioSimCSE-BioLinkBERT-BASE (BioSimCSE) and NeuML/pubmedbert-base-embeddings (PubMedBERT). BioSimCSE builds on BioLinkBERT [26] and was developed for biomedical sentence similarity and retrieval [12]. PubMedBERT provides domain-specific biomedical language representations [11] and the NeuML checkpoint adapts it for sentence-level semantic search and similarity.

For bi-encoder retrieval, query and CDE texts were encoded separately and candidate CDEs were ranked by cosine similarity. Model and text-representation selection were performed on the Validation split, using Recall@5 as the primary criterion and Recall@10 and MRR@100 as secondary ranking-sensitive criteria. The internal test set and the distribution-shifted holdout sets were never used for tuning, model selection, or checkpoint selection; they were evaluated only after validation-based selection was complete, using the final selected version of each model.

The same selection procedure was applied at Phase 1 and Phase 2. Each complete hyperparameter configuration was run with two seeds (0 and 1) and the two runs were averaged. Configurations were ordered first by mean Validation Recall@5; Recall@10 and then MRR@100 were consulted only to break exact ties in the preceding metric. Every run trained for a fixed number of epochs and used the resulting final checkpoint; no within-run checkpoint selection was performed. A single seed from the winning configuration supplied the checkpoint used downstream.

bioRxiv preprint doi: https://doi.org/10.64898/2026.10.14.758967; this version posted October 20, 2026. The copyright holder for this preprint (which was not certified by peer review) is the author/funder, who has granted bioRxiv a license to display the preprint in perpetuity. It is made available under a CC-BY 4.0 International license.

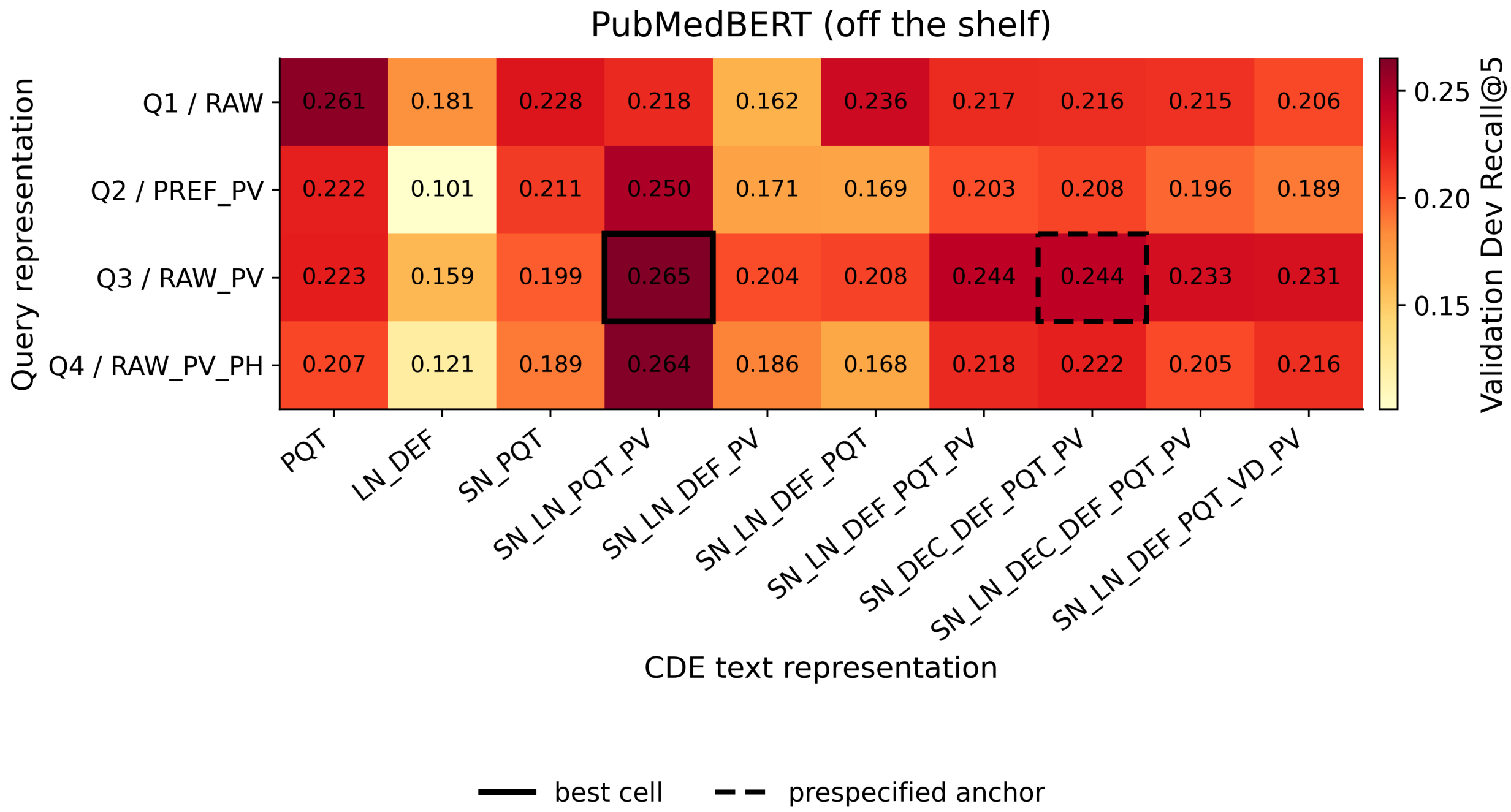

Figure S2: Validation Recall@5 across the same 4 × 10 query–CDE text-representation grid for off-the-shelf PubMedBERT (n = 4,234 query–CDE pairs from 3,934 distinct Validation queries). The solid box marks the best cell and the dashed box the prespecified anchor.

S4.2 Representation-by-loss screening

After fixing text-representation and formatting choices, we screened, for each model, the prespecified anchor representation pair and that model's best-performing non-anchor pair from the off-the-shelf screen, under MNRL [21] and a symmetric bidirectional contrastive variant [22]. Each screening run used 3 epochs, learning rate 5×10^{-5}, temperature 0.05, and two random seeds. The best-performing representation-loss combination was then selected independently for each model and used in that model's main Phase 1 sweep.

S4.3 Phase 1 fine-tuning

The Phase 1 hyperparameter grid was: learning rate $\{7 \times 10^{-5}, 1 \times 10^{-4}, 1.5 \times 10^{-4}\}$, temperature {0.04, 0.07, 0.10}, and epochs {1, 2, 3}, giving 27 configurations per model and, across two seeds, 54 runs per model. Both the representation-and-loss screen and the Phase 1 sweep used a maximum sequence length of 256 tokens, a physical batch size of 128 query-positive pairs - at most 256 encoded strings per optimizer step - and gradient accumulation of 1. Phase 1 executed with float16 autocast, FP32 model parameters, and gradient scaling. To reduce false negatives during in-batch contrastive training, we used a no-duplicate batch sampler that prevented repeated normalized query text or repeated normalized CDE text within the same batch.

At the 256-token fine-tuning limit, query truncation was negligible: only one of the 55,525 distinct fine-tuning queries exceeded the limit for all-MPNet, and none did for BioSimCSE or PubMedBERT. On the CDE side, 940 of 62,976 catalog records (1.49%) exceeded 256 tokens under the SN + DEC + DEF + PQT + PV representation.

S4.4 Phase 2 hard-negative refinement

Phase 2 began from the selected Phase 1 checkpoint for each model and used the same loss family selected in Phase 1. Each configuration was trained for one epoch with two random seeds, a maximum sequence length of 256 tokens, gradient accumulation of 1, and FP32 parameters without autocast or gradient scaling.

To make the negative-mining strategies comparable, each optimizer step encoded 132 text sequences. The condition with no mined negatives used 66 anchor–positive pairs, whereas the mined-negative conditions used 11 training examples, each containing one anchor, one positive, and 10 negatives. Learning-rate and temperature grids were defined relative to the corresponding Phase 1 winner:

bioRxiv preprint doi: https://doi.org/10.64898/2026.10.14.758967; this version posted October 20, 2026. The copyright holder for this preprint (which was not certified by peer review) is the author/funder, who has granted bioRxiv a license to display the preprint in perpetuity. It is made available under a CC-BY 4.0 International license.

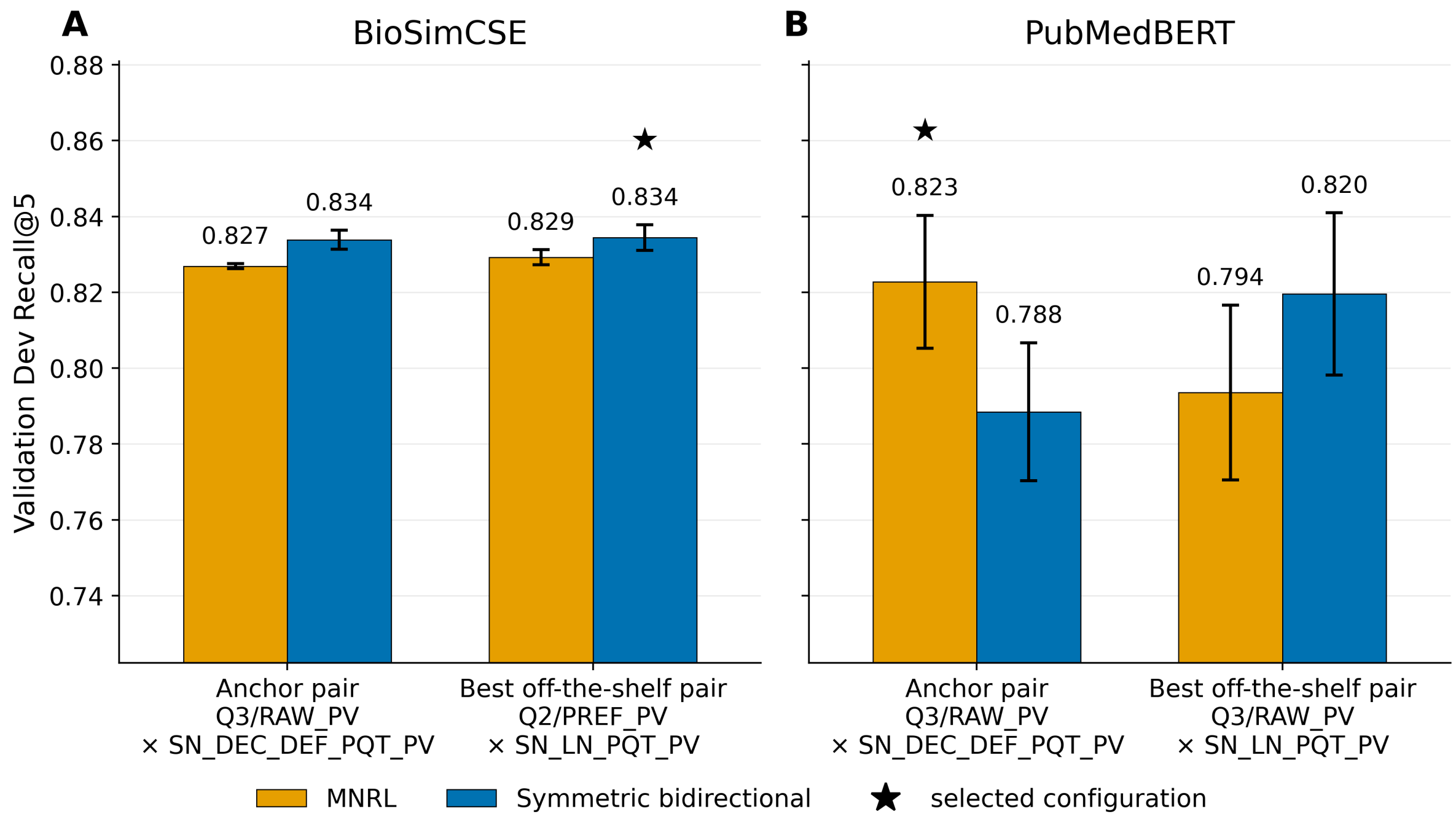

Figure S3: Validation Recall@5 for BioSimCSE (left) and PubMedBERT (right) across the two representation pairs and the MNRL and symmetric bidirectional losses, on a shared scale. Bars show the two-seed mean and error bars the sample standard deviation; the selected condition is marked.

- $7 \times 10^{-5} \to \{3 \times 10^{-5}, 5 \times 10^{-5}, 7 \times 10^{-5}\}$
- $1 \times 10^{-4} \to \{5 \times 10^{-5}, 7 \times 10^{-5}, 1 \times 10^{-4}\}$
- $1.5 \times 10^{-4} \to \{7 \times 10^{-5}, 1 \times 10^{-4}, 1.5 \times 10^{-4}\}$

The temperature mappings were:

- $0.04 \to \{0.03, 0.04, 0.05\}$
- $0.07 \to \{0.04, 0.06, 0.08\}$
- $0.10 \to \{0.07, 0.08, 0.10\}$

We compared three strategies: no mined negatives, hard negatives sampled from ranks 1–25, and semi-hard negatives sampled from ranks 1–50. The negative-mining strategy was treated as part of the hyperparameter configuration, and configurations were selected using the same Validation procedure described in Section S4.1.

FT-MPNet was selected as the final bi-encoder for downstream candidate generation and reranking. Where Phase 2 results for the biomedical bi-encoders are reported, they are descriptive; differences of the observed magnitude fall within seed-to-seed variability and are not interpreted as evidence of statistical superiority.

S4.5 Bi-encoder performance

Figure S4 summarizes Validation performance across off-the-shelf inference, Phase 1 fine-tuning, and Phase 2 refinement. Table S4 reports the retained Phase 2 models on the six evaluation datasets and includes their Validation selection results for reference.

Table S4. Bi-encoder Recall@5 on the six canonical evaluation datasets. Each cell is the mean over the two seeds of that model's winning Phase 2 configuration, with the sample standard deviation; evaluation is inference-only on the frozen checkpoints, query-level against reachable gold, with public-identifier deduplication, over the same June production catalog (62,976 CDEs).

Dataset	all-MPNet	BioSimCSE	PubMedBERT
Test	0.9089 ± 0.0038	0.8991 ± 0.0034	0.9059 ± 0.0005
CCTG	0.7019 ± 0.0039	0.6860 ± 0.0058	0.6878 ± 0.0032
OID ALT	0.4391 ± 0.0156	0.4108 ± 0.0052	0.3884 ± 0.0088

bioRxiv preprint doi: https://doi.org/10.64898/2026.10.14.758967; this version posted October 20, 2026. The copyright holder for this preprint (which was not certified by peer review) is the author/funder, who has granted bioRxiv a license to display the preprint in perpetuity. It is made available under a CC-BY 4.0 International license.

Dataset	all-MPNet	BioSimCSE	PubMedBERT
CDASH	0.8210 ± 0.0262	0.8133 ± 0.0240	0.8272 ± 0.0000
GDC	0.8542 ± 0.0295	0.8333 ± 0.0196	0.8611 ± 0.0196
CIMAC	0.5611 ± 0.0054	0.4809 ± 0.0324	0.5191 ± 0.0108
Validation (Phase 2 selection)	0.9014 ± 0.0008	0.8941 ± 0.0002	0.8966 ± 0.0000

For CIMAC, which was assembled independently of caDSR, the Q2 prefix reflects its ALT-style classification (Section 2.2) rather than caDSR metadata provenance.

S5. Baselines and hybrid reranking system

S5.1 BM25 baseline

BM25 was Okapi BM25 with the standard Lucene non-negative inverse-document-frequency formulation, computed over unigram term counts. The weighting constants were left at their conventional values of k1 = 1.2 and b = 0.75 and were never tuned; query terms were weighted binarily, so a repeated query term contributes once. Documents and queries were tokenized identically: Unicode normalization, case folding, and splitting on runs of alphanumeric and underscore characters. No stop-word list, stemming, lemmatization or n-gram expansion was applied, and no vocabulary pruning was used. Fields absent for a given CDE were omitted rather than replaced by a placeholder.

BM25 retrieved from the same production CDE catalog as the neural methods and was evaluated on the same six canonical datasets with the same reachable-gold filtering, query-level denominators, and public-identifier-level gold matching. Retrieved CDEs were deduplicated by public identifier, retaining the highest-scoring version, so no public identifier can occupy more than one rank for a query. Ties in the BM25 score were broken deterministically by catalog order. Because BM25 has no learned parameters and no random initialization, the procedure is fully deterministic and was executed once; no seed average or standard deviation is reported for it.

For BM25 we swept the same canonical grid of four query representations by ten CDE text recipes used elsewhere in this work, evaluating every cell on Validation and freezing the best-performing cell before any evaluation set was touched. Selection ranked cells by Validation Recall@5 and then MRR@100. The selected configuration pairs the raw query text with its permissible-value summary (Q3) with the SN + LN + PQT + PV CDE recipe, which concatenates short name, long name, preferred question text and permissible-value summary in labelled form. Test and the distribution-shifted holdout sets played no part in this selection.

S5.2 CDE Match-Fuzzy keyword retrieval

CDE Match-Fuzzy searches CDE metadata with the exact, like and reverse-like rules of the documented matching flow, and adds character-n-gram and token-overlap fuzzy signals over DEC long name, long name, preferred question text. Each rule contributes candidates to a per-rule depth of 500, the fuzzy stage retains its top 1000 candidates, and the merged, deduplicated list is truncated to the top 10 candidates that enter the candidate pool. Evidence from several rules is aggregated per candidate, candidates are deduplicated by CDE public identifier, and exactly equal scores are broken deterministically by ascending public identifier.

Candidate depth. CDE Match-Fuzzy used a per-rule depth of 500 for the six evaluation datasets and 100 for Validation selection before truncation to the top 10 merged candidates. On Validation, the two settings produced identical top-10 lists for 99.6% of queries and no difference in gold inclusion at ranks 5, 10 or 20; all final feature evidence used depth 500.

Eligibility. Retrieval is restricted to the frozen production CDE catalog (62,976 CDEs; eligibility mode production_cde_match), which excludes retired, archived and non-production administrative records.

S5.3 Candidate-pool construction and selection of K

The reranker operates on a bounded candidate pool rather than the full catalog. For each query, candidates retrieved by FT-MPNet were combined with candidates from the keyword/fuzzy retriever, and the union was deduplicated by CDE public identifier. The nominal pool size K is the sum of the two retrieval depths. To choose K we held the keyword/fuzzy depth fixed at its deployed value of 10 and swept the bi-encoder depth, giving nominal

bioRxiv preprint doi: https://doi.org/10.64898/2026.10.14.758967; this version posted October 20, 2026. The copyright holder for this preprint (which was not certified by peer review) is the author/funder, who has granted bioRxiv a license to display the preprint in perpetuity. It is made available under a CC-BY 4.0 International license.

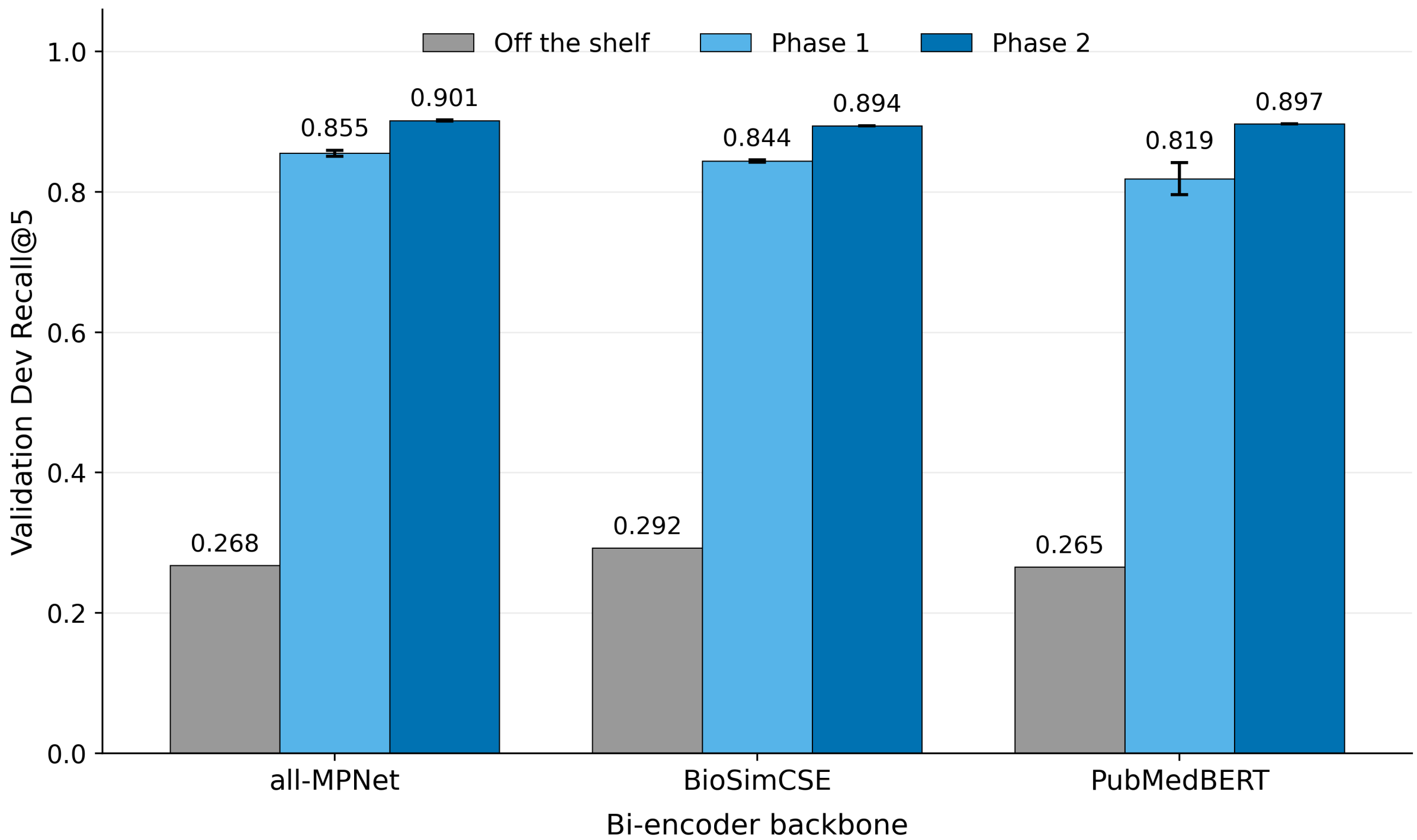

Figure S4: Validation Recall@5 for all-MPNet, BioSimCSE and PubMedBERT across off-the-shelf inference, Phase 1 fine-tuning and Phase 2 refinement. Fine-tuned stages show the two-seed mean with the sample standard deviation; off-the-shelf inference is deterministic and is shown without an error bar.

pool sizes of K = 20, 30, 40, and 60. Because the two retrievers frequently return the same CDE, realized pools are often smaller than nominal K: at K = 30 the mean realized pool contains 27.6 candidates, and roughly 88-90% of queries yield fewer than 30 distinct candidates.

Pools were compared using ceiling Recall@5, defined as the proportion of queries for which at least one accepted gold CDE was present anywhere in the candidate pool. Because Recall@5 is hit-based, a gold CDE present anywhere in the pool could in principle be placed within the top five by an ideal reranker, so this quantity is equivalently the maximum Recall@5 achievable by an oracle reranker operating on that pool. It therefore bounds the performance of any reranker built on the pool and is independent of the reranker itself.

K was selected on Reranker Training; Validation was used only as descriptive confirmation and played no part in the choice. Ceiling Recall@5 rises from 0.9828 at K = 20 to 0.9888 at K = 30 on Reranker Training and then flattens, gaining only a further 0.0036 between K = 30 and K = 60 while roughly doubling the pool. We selected K = 30, at the onset of this plateau, as a coverage-versus-computation trade-off; Validation gives the same ceiling Recall@5 of 0.9888 at K = 30. This is a practical operating point rather than a statistically identified elbow, and no significance claim is made.

S5.4 Cross-encoder training and selection

Three cross-encoder backbones were compared: ncbi/MedCPT-Cross-Encoder, BAAI/bge-reranker-base, cross-encoder/ms-marco-MiniLM-L-6-v2. Training pairs were drawn from the corrected, production-eligible candidate pool, so no candidate carried empty text and no ineligible CDE was scored.

All backbones were trained under one frozen protocol (BCE pointwise, 2 epochs, learning rate 2e-5, batch size 32, maximum length 512, seed 20260527, trained on the full train split), with the positive class weighted by the ratio of negative to positive pairs, and were compared on Validation by Recall@5. FT-MedCPT was selected with Validation Recall@5 0.912, ahead of FT-BGE (0.904) and FT-MiniLM (0.874).

bioRxiv preprint doi: https://doi.org/10.64898/2026.10.14.758967; this version posted October 20, 2026. The copyright holder for this preprint (which was not certified by peer review) is the author/funder, who has granted bioRxiv a license to display the preprint in perpetuity. It is made available under a CC-BY 4.0 International license.

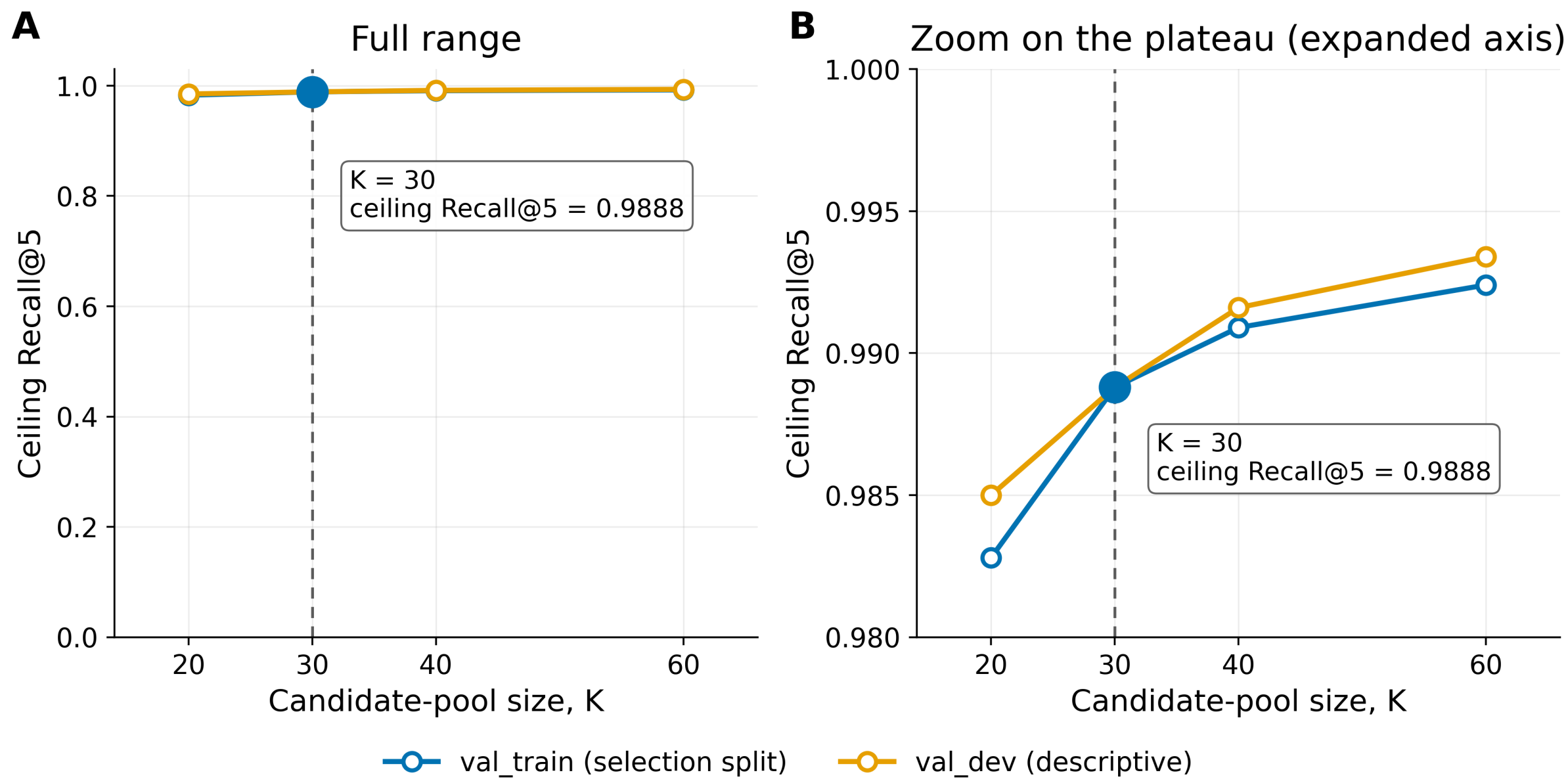

Figure S5: Ceiling Recall@5 as a function of nominal candidate-pool size K. Pools combine fine-tuned MPNet retrieval with a fixed keyword/fuzzy depth of 10. K was selected on Reranker Training; Validation is descriptive confirmation only. K = 30 was selected.

S5.5 HGBC reranker

The final ranking model was a histogram-based gradient-boosting classifier (HGBC) implemented in scikit-learn. For each query, it scored the union of the top 20 FT-MPNet candidates and the top 10 CDE Match-Fuzzy candidates after deduplication by CDE public identifier. This produced a candidate pool of at most 30 CDEs per query, with a mean size of approximately 28. Candidate pools were constructed in the same way for training, validation, and evaluation, without adding the gold CDE when it was absent.

The HGBC was trained on the Reranker Training split, which contained 3,946 queries. Together, their candidate pools yielded 109,141 query–candidate pairs. A pair was labeled positive when the candidate's public identifier matched any accepted gold CDE for that query; all other pairs were labeled negative. The training data contained 4,109 positive pairs, approximately 3.7% of the total. Queries with multiple accepted gold CDEs could contribute more than one positive pair. No negative sampling, class weighting, or sample weighting was used.

Each query–candidate pair was represented by 117 features in seven groups: retrieval-source indicators; FT-MPNet scores and ranks; keyword exact- and synonym-tier scores; permissible-value overlap; query and CDE text characteristics; keyword-rule evidence; and FT-MedCPT scores and ranks. Features that were not defined for a particular candidate—for example, a keyword score for a candidate retrieved only by FT-MPNet—were retained as missing values. The HGBC handles these values directly by learning, at each tree split, which branch missing values should follow. No global feature normalization or categorical features were used in the final model.

Hyperparameters were selected from 16 combinations of boosting iterations {200, 500}, maximum tree depth {3, 5}, learning rate {0.05, 0.1}, and minimum samples per leaf {10, 30}. Selection used Recall@5 on Validation, and no test or external evaluation dataset was used for training or model selection. The selected configuration used 200 boosting iterations, maximum depth 3, learning rate 0.05, minimum samples per leaf 30, and random seed 42. A prespecified follow-up comparison selected the feature set without query-provenance variables. Content-level training–evaluation overlap is characterized separately in Section S1.5 and Table S6.

At inference, candidates were ranked within each query by their predicted probability of being a gold CDE. Exact score ties were broken by ascending CDE public identifier.

S5.6 Candidate-pool coverage and retrieval complementarity

Table S5. Candidate-pool gold coverage by retrieval arm. Coverage is the proportion of queries whose accepted gold CDE occurs anywhere in the stated candidate set, matched at the CDE public-identifier level; n is the

bioRxiv preprint doi: https://doi.org/10.64898/2026.10.14.758967; this version posted October 20, 2026. The copyright holder for this preprint (which was not certified by peer review) is the author/funder, who has granted bioRxiv a license to display the preprint in perpetuity. It is made available under a CC-BY 4.0 International license.

denominator. The union is the fixed candidate pool used by the final reranker.

Dataset	n	FT-MPNet@20	CDE Match-Fuzzy@10	Union
Test	3,959	0.958	0.795	0.986
CCTG	1,097	0.841	0.807	0.959
OID ALT	1,766	0.555	0.710	0.865
CDASH	324	0.923	0.861	0.969
GDC	72	0.917	0.972	0.986
CIMAC	131	0.672	0.809	0.870

Coverage differed by retrieval method and dataset, but their union improved on the better individual retriever in every dataset, reaching 0.865–0.986.

S5.7 Contribution of feature groups to final ranking

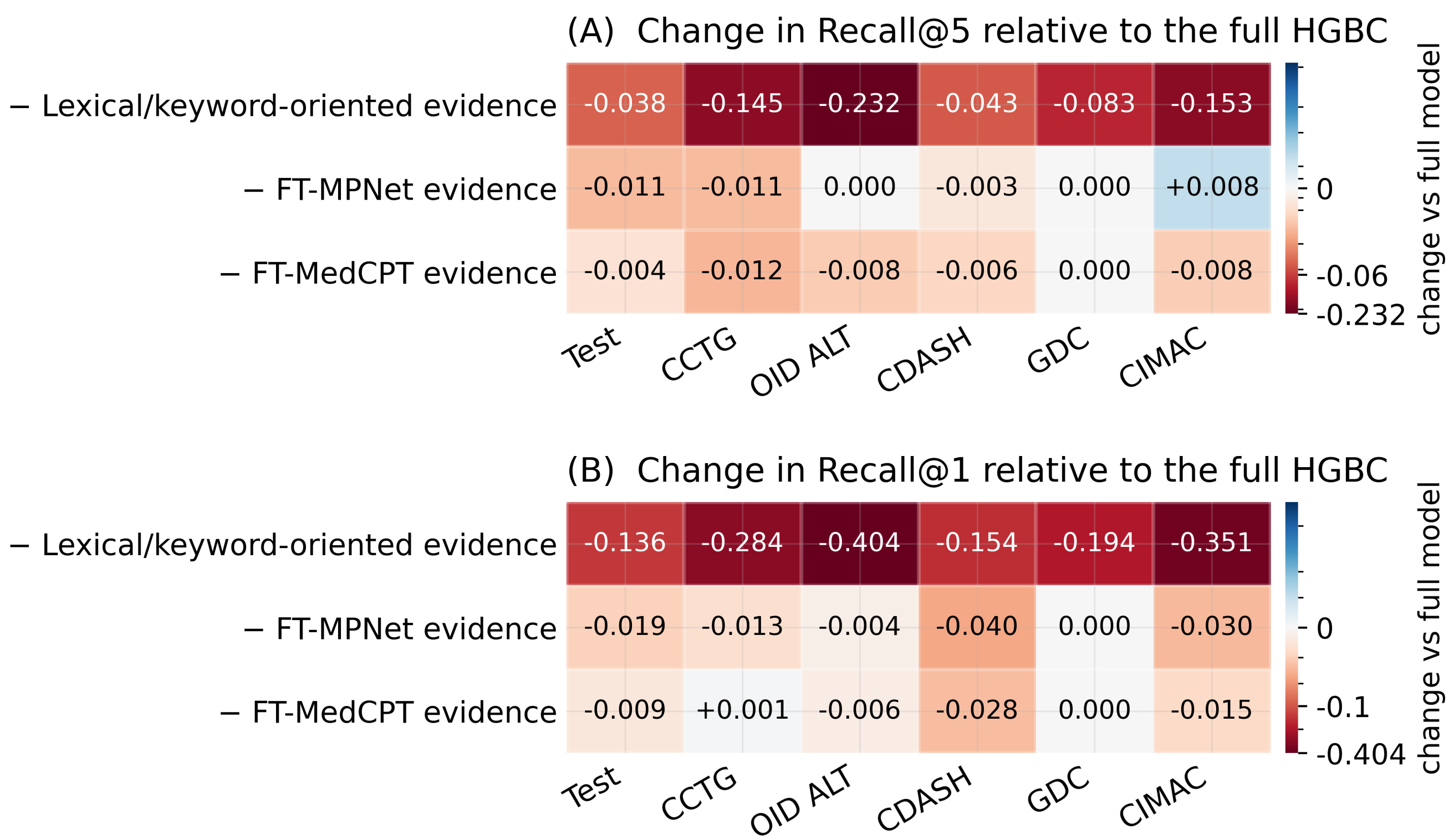

Figure S6: Broad evidence-family ablation of the final HGBC. Change in Recall@5 (A) and Recall@1 (B) after retraining the HGBC with one of three evidence families removed: lexical/keyword-oriented (98 features), FT-MPNet (13) and FT-MedCPT (6), which together partition all 117 features. All conditions use the identical fixed Stage-1 candidate pool, so the experiment measures contribution to ranking within the pool rather than to candidate generation. Cells give the change from the full 117-feature model; negative values (red) indicate lower recall. The colour scale is centred at zero and compressed beyond the largest neural-family effect so that the smaller effects remain visible; exact values are printed in every cell.

To isolate the evidence used for final ranking from candidate generation, we retrained the HGBC after removing each of three feature families while holding the Stage-1 candidate pool fixed. Removing lexical/keyword-oriented evidence produced the largest losses across datasets, whereas removing FT-MPNet or FT-MedCPT evidence produced smaller, dataset-dependent changes. This suggests that once a set of plausible candidates has been retrieved, detailed lexical cues such as identifiers, abbreviations, qualifiers, token overlap, and permissible-value agreement are especially useful for distinguishing the correct CDE. The lexical family contains multiple overlapping signals, so its contribution reflects their combined effect rather than any single keyword feature.

bioRxiv preprint doi: https://doi.org/10.64898/2026.10.14.758967; this version posted October 20, 2026. The copyright holder for this preprint (which was not certified by peer review) is the author/funder, who has granted bioRxiv a license to display the preprint in perpetuity. It is made available under a CC-BY 4.0 International license.

S6. Sensitivity and robustness analyses

S6.1 Training-evaluation overlap sensitivity

Table S6 evaluates whether removing repeated training–evaluation mappings changes the reported method performance.

Table S6. Sensitivity of Recall@5 to removing external-evaluation queries whose normalized text and gold CDE both recur in training. Filtered values are computed by rescoring the same saved rankings against the leakage-filtered query lists. The internal Test split is in-distribution by design and is not included.

Dataset	Method	n (all)	Recall@5 (all)	n (filtered)	Recall@5 (filtered)	Δ
CCTG	Final reranker	1,097	0.909	1,089	0.909	+0.0002
CCTG	Python approximation to NCI CDE Match	1,097	0.739	1,089	0.738	-0.0010
CCTG	BM25	1,097	0.392	1,089	0.394	+0.0020
OID ALT	Final reranker	1,766	0.832	1,759	0.832	-0.0007
OID ALT	Python approximation to NCI CDE Match	1,766	0.770	1,759	0.769	-0.0009
OID ALT	BM25	1,766	0.176	1,759	0.176	+0.0001
CDASH	Final reranker	324	0.920	310	0.919	-0.0004
CDASH	Python approximation to NCI CDE Match	324	0.750	310	0.748	-0.0016
CDASH	BM25	324	0.710	310	0.729	+0.0192
GDC	Final reranker	72	0.972	68	0.971	-0.0016
GDC	Python approximation to NCI CDE Match	72	0.986	68	0.985	-0.0008
GDC	BM25	72	0.542	68	0.515	-0.0270
CIMAC	Final reranker	131	0.802	113	0.779	-0.0228
CIMAC	Python approximation to NCI CDE Match	131	0.679	113	0.637	-0.0422
CIMAC	BM25	131	0.160	113	0.150	-0.0099

S6.2 Exact-match allowance sensitivity

Both lexical methods used the same deterministic query-level mask to limit direct exact lookup of the gold CDE. The exact-match rules search the CDE long name, question-text strings in its reference documents—including preferred and alternate question text—and alternate names. A query harvested from one gold-linked metadata field can be identical to a string stored in another, so the mask covers all exact-match-eligible fields rather than only the field from which the query originated.

bioRxiv preprint doi: https://doi.org/10.64898/2026.10.14.758967; this version posted October 20, 2026. The copyright holder for this preprint (which was not certified by peer review) is the author/funder, who has granted bioRxiv a license to display the preprint in perpetuity. It is made available under a CC-BY 4.0 International license.

Queries were assigned to nested allowance subsets using a SHA-1 hash of the query identifier with seed 42. For a masked query, only strings belonging to the gold CDE that were identical to the normalized query were withheld. The query itself, other catalog entries, the gold CDE's nonidentical metadata, non-exact matching rules, and permissible values were unchanged. The Python approximation to NCI CDE Match and CDE Match-Fuzzy therefore received the same exact-match availability at every allowance rate.

The 70% operating rate was motivated by CIMAC, where 92 of 131 queries whose gold CDE was present in the catalog had an exact match when all eligible metadata were available (70.2%). We therefore used a 70% allowance for the four caDSR-derived evaluation sets and a 100% allowance for GDC and CIMAC, which were assembled independently and do not create the same circularity.

The fixed HGBC trained at the 70% allowance was substantially less sensitive to allowance changes than either lexical method. Across the full 0-100% range its Recall@5 varied by 0.075 on Test and 0.164 on CDASH, whereas the two lexical methods varied by 0.40-0.96 on every dataset. At the 0% allowance, where every exact-matching string of the gold CDE was withheld, the fixed reranker still reached 0.920 on Test and 0.818 on CDASH, compared with 0.151-0.556 for the lexical methods. Its performance also remained close to that of HGBC models retrained separately at each allowance, differing by at most 0.02 Recall@5 in 20 of 24 dataset-rate conditions; the largest difference was on CDASH at the 0% allowance, where retraining recovered 0.065 Recall@5. Retraining therefore provided limited additional benefit under most conditions rather than none. OID ALT remained the most allowance-sensitive dataset, with Recall@5 falling from 0.832 at the 70% allowance to 0.463 at 0%. Much of that loss was a limit of candidate generation rather than of ranking: the proportion of OID ALT queries whose accepted gold CDE entered the candidate pool at all fell from 0.865 at the 70% allowance to 0.571 at 0%, where 758 of 1,766 gold CDEs were absent from the pool. No reranker can recover a gold CDE that is not in the pool. At the 100% allowance all methods converged near ceiling, so that condition is an upper anchor rather than a discriminating comparison.

Table S7. Exact-match allowance sensitivity for the Python approximation to NCI CDE Match, CDE Match-Fuzzy, and the fixed HGBC trained at 70% allowance: Recall@1, Recall@5, Recall@10, and MRR@100.

Dataset	Allowance rate	Method	Recall@1	Recall@5	Recall@10	MRR@100
Test	0%	Python approximation to NCI CDE Match	0.071	0.151	0.173	0.103
Test	0%	CDE Match-Fuzzy	0.166	0.325	0.394	0.243
Test	0%	Fixed HGBC trained at 70%	0.734	0.920	0.950	0.816
Test	50%	Python approximation to NCI CDE Match	0.506	0.562	0.574	0.530
Test	50%	CDE Match-Fuzzy	0.550	0.646	0.680	0.597
Test	50%	Fixed HGBC trained at 70%	0.853	0.959	0.973	0.899
Test	60%	Python approximation to NCI CDE Match	0.588	0.642	0.652	0.611
Test	60%	CDE Match-Fuzzy	0.622	0.713	0.742	0.665
Test	60%	Fixed HGBC trained at 70%	0.873	0.966	0.977	0.914

bioRxiv preprint doi: https://doi.org/10.64898/2026.10.14.758967; this version posted October 20, 2026. The copyright holder for this preprint (which was not certified by peer review) is the author/funder, who has granted bioRxiv a license to display the preprint in perpetuity. It is made available under a CC-BY 4.0 International license.

Dataset	Allowance rate	Method	Recall@1	Recall@5	Recall@10	MRR@100
Test	70%	Python approximation to NCI CDE Match	0.666	0.719	0.727	0.689
Test	70%	CDE Match-Fuzzy	0.690	0.773	0.795	0.729
Test	70%	Fixed HGBC trained at 70%	0.896	0.971	0.980	0.929
Test	80%	Python approximation to NCI CDE Match	0.759	0.807	0.813	0.779
Test	80%	CDE Match-Fuzzy	0.773	0.843	0.858	0.805
Test	80%	Fixed HGBC trained at 70%	0.925	0.981	0.987	0.950
Test	100%	Python approximation to NCI CDE Match	0.926	0.963	0.965	0.942
Test	100%	CDE Match-Fuzzy	0.920	0.963	0.968	0.939
Test	100%	Fixed HGBC trained at 70%	0.970	0.995	0.997	0.981
CCTG	0%	Python approximation to NCI CDE Match	0.065	0.130	0.159	0.090
CCTG	0%	CDE Match-Fuzzy	0.143	0.281	0.350	0.211
CCTG	0%	Fixed HGBC trained at 70%	0.478	0.722	0.796	0.585
CCTG	50%	Python approximation to NCI CDE Match	0.522	0.576	0.593	0.545
CCTG	50%	CDE Match-Fuzzy	0.562	0.654	0.694	0.607
CCTG	50%	Fixed HGBC trained at 70%	0.722	0.868	0.905	0.786
CCTG	60%	Python approximation to NCI CDE Match	0.600	0.652	0.666	0.621
CCTG	60%	CDE Match-Fuzzy	0.629	0.711	0.747	0.670
CCTG	60%	Fixed HGBC trained at 70%	0.755	0.883	0.915	0.812

bioRxiv preprint doi: https://doi.org/10.64898/2026.10.14.758967; this version posted October 20, 2026. The copyright holder for this preprint (which was not certified by peer review) is the author/funder, who has granted bioRxiv a license to display the preprint in perpetuity. It is made available under a CC-BY 4.0 International license.

Dataset	Allowance rate	Method	Recall@1	Recall@5	Recall@10	MRR@100
CCTG	70%	Python approximation to NCI CDE Match	0.686	0.739	0.753	0.709
CCTG	70%	CDE Match-Fuzzy	0.703	0.780	0.807	0.741
CCTG	70%	Fixed HGBC trained at 70%	0.798	0.909	0.934	0.847
CCTG	80%	Python approximation to NCI CDE Match	0.759	0.812	0.823	0.782
CCTG	80%	CDE Match-Fuzzy	0.774	0.846	0.867	0.808
CCTG	80%	Fixed HGBC trained at 70%	0.846	0.933	0.949	0.884
CCTG	100%	Python approximation to NCI CDE Match	0.914	0.974	0.985	0.941
CCTG	100%	CDE Match-Fuzzy	0.914	0.974	0.986	0.941
CCTG	100%	Fixed HGBC trained at 70%	0.931	0.988	0.993	0.956
OID ALT	0%	Python approximation to NCI CDE Match	0.168	0.252	0.275	0.205
OID ALT	0%	CDE Match-Fuzzy	0.015	0.040	0.063	0.032
OID ALT	0%	Fixed HGBC trained at 70%	0.299	0.463	0.528	0.373
OID ALT	50%	Python approximation to NCI CDE Match	0.563	0.614	0.625	0.586
OID ALT	50%	CDE Match-Fuzzy	0.486	0.506	0.519	0.499
OID ALT	50%	Fixed HGBC trained at 70%	0.623	0.716	0.753	0.666
OID ALT	60%	Python approximation to NCI CDE Match	0.643	0.690	0.696	0.663
OID ALT	60%	CDE Match-Fuzzy	0.581	0.603	0.613	0.594
OID ALT	60%	Fixed HGBC trained at 70%	0.691	0.776	0.807	0.730

bioRxiv preprint doi: https://doi.org/10.64898/2026.10.14.758967; this version posted October 20, 2026. The copyright holder for this preprint (which was not certified by peer review) is the author/funder, who has granted bioRxiv a license to display the preprint in perpetuity. It is made available under a CC-BY 4.0 International license.

Dataset	Allowance rate	Method	Recall@1	Recall@5	Recall@10	MRR@100
OID ALT	70%	Python approximation to NCI CDE Match	0.728	0.770	0.775	0.746
OID ALT	70%	CDE Match-Fuzzy	0.682	0.702	0.710	0.693
OID ALT	70%	Fixed HGBC trained at 70%	0.764	0.832	0.853	0.796
OID ALT	80%	Python approximation to NCI CDE Match	0.812	0.847	0.850	0.827
OID ALT	80%	CDE Match-Fuzzy	0.782	0.802	0.805	0.793
OID ALT	80%	Fixed HGBC trained at 70%	0.837	0.887	0.902	0.862
OID ALT	100%	Python approximation to NCI CDE Match	0.981	0.999	0.999	0.989
OID ALT	100%	CDE Match-Fuzzy	0.982	1.000	1.000	0.990
OID ALT	100%	Fixed HGBC trained at 70%	0.977	1.000	1.000	0.988
CDASH	0%	Python approximation to NCI CDE Match	0.093	0.238	0.284	0.157
CDASH	0%	CDE Match-Fuzzy	0.318	0.556	0.617	0.430
CDASH	0%	Fixed HGBC trained at 70%	0.574	0.818	0.883	0.685
CDASH	50%	Python approximation to NCI CDE Match	0.472	0.599	0.630	0.528
CDASH	50%	CDE Match-Fuzzy	0.568	0.762	0.809	0.654
CDASH	50%	Fixed HGBC trained at 70%	0.750	0.892	0.926	0.816
CDASH	60%	Python approximation to NCI CDE Match	0.537	0.664	0.694	0.591
CDASH	60%	CDE Match-Fuzzy	0.614	0.796	0.836	0.693
CDASH	60%	Fixed HGBC trained at 70%	0.781	0.910	0.941	0.839

bioRxiv preprint doi: https://doi.org/10.64898/2026.10.14.758967; this version posted October 20, 2026. The copyright holder for this preprint (which was not certified by peer review) is the author/funder, who has granted bioRxiv a license to display the preprint in perpetuity. It is made available under a CC-BY 4.0 International license.

Dataset	Allowance rate	Method	Recall@1	Recall@5	Recall@10	MRR@100
CDASH	70%	Python approximation to NCI CDE Match	0.633	0.750	0.775	0.680
CDASH	70%	CDE Match-Fuzzy	0.673	0.830	0.861	0.740
CDASH	70%	Fixed HGBC trained at 70%	0.809	0.920	0.944	0.858
CDASH	80%	Python approximation to NCI CDE Match	0.698	0.815	0.840	0.746
CDASH	80%	CDE Match-Fuzzy	0.722	0.886	0.910	0.790
CDASH	80%	Fixed HGBC trained at 70%	0.843	0.938	0.957	0.883
CDASH	100%	Python approximation to NCI CDE Match	0.840	0.960	0.975	0.889
CDASH	100%	CDE Match-Fuzzy	0.840	0.960	0.975	0.890
CDASH	100%	Fixed HGBC trained at 70%	0.907	0.981	0.985	0.938

bioRxiv preprint doi: https://doi.org/10.64898/2026.10.14.758967; this version posted October 20, 2026. The copyright holder for this preprint (which was not certified by peer review) is the author/funder, who has granted bioRxiv a license to display the preprint in perpetuity. It is made available under a CC-BY 4.0 International license.

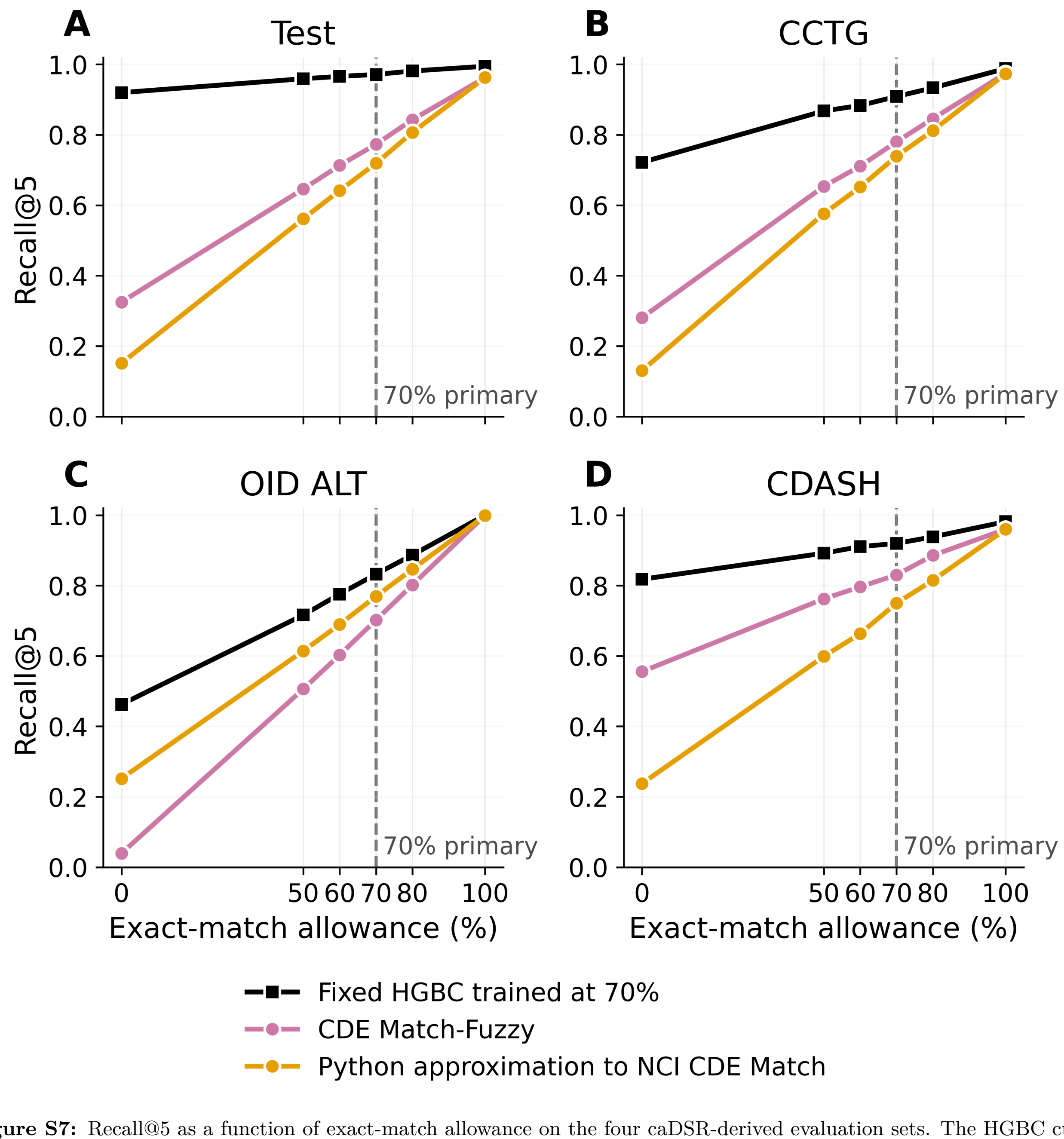

Figure S7: Recall@5 as a function of exact-match allowance on the four caDSR-derived evaluation sets. The HGBC curve uses the unchanged reranker trained at the 70% allowance. At each inference allowance, CDE Match-Fuzzy candidates, merged candidate pools, and allowance-dependent features were regenerated, while FT-MPNet, FT-MedCPT, and HGBC model weights remained fixed. The 70% operating point used in the primary analysis is marked.

bioRxiv preprint doi: https://doi.org/10.64898/2026.10.14.758967; this version posted October 20, 2026. The copyright holder for this preprint (which was not certified by peer review) is the author/funder, who has granted bioRxiv a license to display the preprint in perpetuity. It is made available under a CC-BY 4.0 International license.

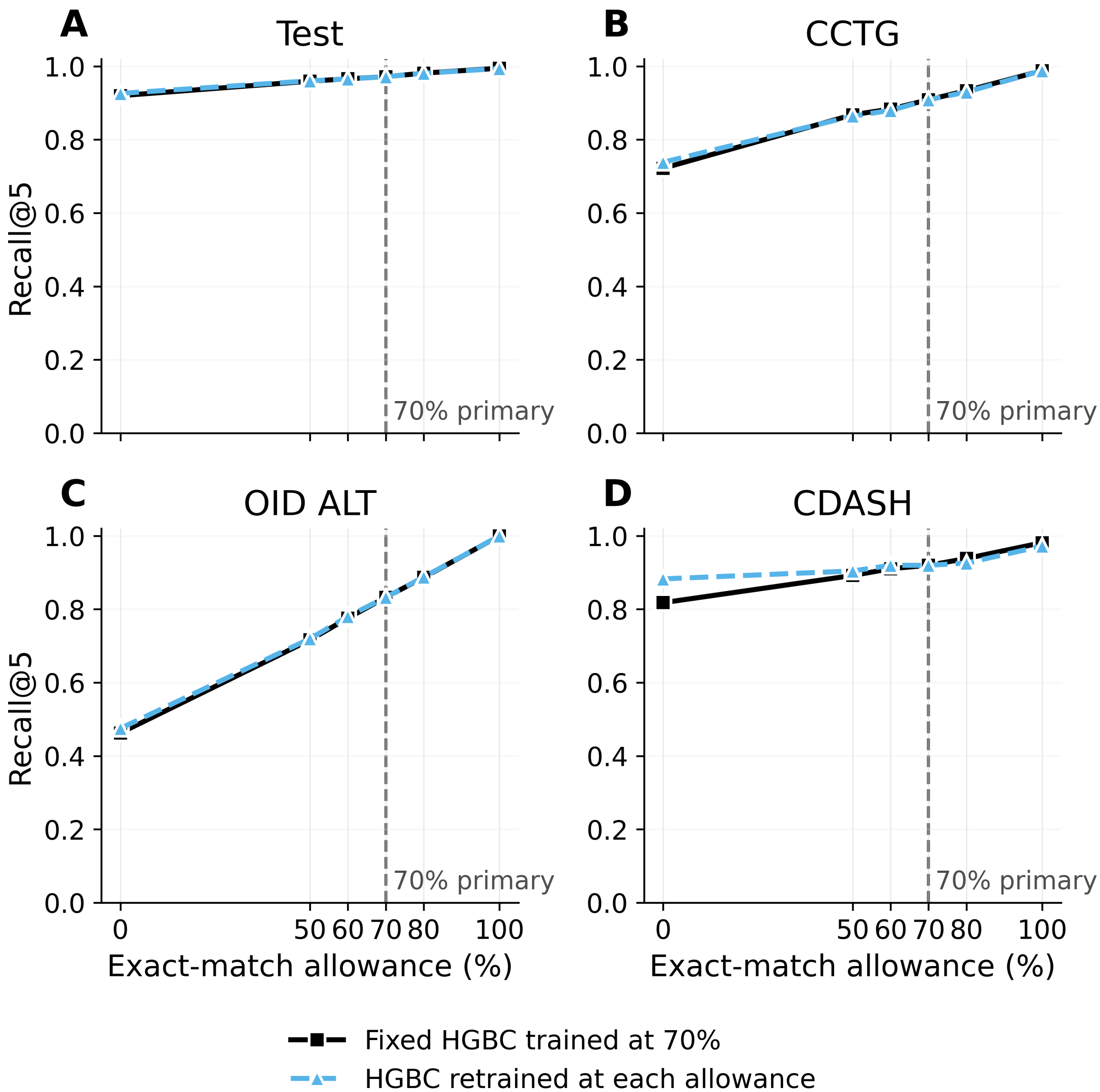

Figure S8: Recall@5 for the fixed HGBC trained at the 70% allowance and HGBC models retrained separately at each allowance. Candidate pools and allowance-dependent features were generated at the indicated allowance for both analyses; FT-MPNet and FT-MedCPT remained fixed. The fixed-model analysis represents inference-time mismatch in a deployed pipeline, whereas the retrained analysis measures adaptation of the HGBC training procedure.
